# Natural Melanin/Alginate Hydrogels Achieve Cardiac Repair through ROS Scavenging and Macrophage Polarization

**DOI:** 10.1002/advs.202100505

**Published:** 2021-08-19

**Authors:** Jin Zhou, Wei Liu, Xiaoyi Zhao, Yifan Xian, Wei Wu, Xiao Zhang, Nana Zhao, Fu‐Jian Xu, Changyong Wang

**Affiliations:** ^1^ Beijing Institute of Basic Medical Sciences 27 Taiping Rd Beijing 100850 P. R. China; ^2^ Key Lab of Biomedical Materials of Natural Macromolecules (Beijing University of Chemical Technology Ministry of Education) Beijing Laboratory of Biomedical Materials Beijing Advanced Innovation Center for Soft Matter Science and Engineering College of Materials Science and Engineering Beijing University of Chemical Technology Beijing 100029 P. R. China

**Keywords:** alginate, cardiac repair, macrophage polarization, melanin nanoparticles, ROS scavenging

## Abstract

The efficacy of cardiac regenerative strategies for myocardial infarction (MI) treatment is greatly limited by the cardiac microenvironment. The combination of reactive oxygen species (ROS) scavenging to suppress the oxidative stress damage and macrophage polarization to regenerative M2 phenotype in the MI microenvironment can be desirable for MI treatment. Herein, melanin nanoparticles (MNPs)/alginate (Alg) hydrogels composed of two marine‐derived natural biomaterials, MNPs obtained from cuttlefish ink and alginate extracted from ocean algae, are proposed. Taking advantage of the antioxidant property of MNPs and mechanical support from injectable alginate hydrogels, the MNPs/Alg hydrogel is explored for cardiac repair by regulating the MI microenvironment. The MNPs/Alg hydrogel is found to eliminate ROS against oxidative stress injury of cardiomyocytes. More interestingly, the macrophage polarization to regenerative M2 macrophages can be greatly promoted in the presence of MNPs/Alg hydrogel. An MI rat model is utilized to evaluate the feasibility of the as‐prepared MNPs/Alg hydrogel for cardiac repair in vivo. The antioxidant, anti‐inflammatory, and proangiogenesis effects of the hydrogel are investigated in detail. The present study opens up a new way to utilize natural biomaterials for MI treatment and allows to rerecognize the great value of natural biomaterials in cardiac repair.

## Graphical abstract

Natural melanin nanoparticles/alginate hydrogels are proposed to attenuate inflammation by scavenging harmful reactive oxygen species and promoting macrophage polarization to regenerative M2 phenotype in the MI region for cardiac repair.

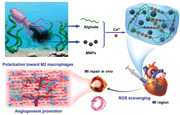

## 1 Introduction

Myocardial infarction (MI) presents high mortality and morbidity around the world, which is usually known as heart attack and occurs upon coronary artery occlusion.^[^ ^1^, ^2^ ^]^ Up to 10–40% of MI can cause heart failure, which is a common complication of MI. The low blood supply and oxygen deprivation at the downstream myocardium caused by MI could induce the apoptosis and necrosis of cardiomyocytes (CMs), which will lead to heart failure. In addition, heart fibroblasts will be activated by MI injury to form noncontractile scar tissues. Traditional interventional or drug treatments face great challenges such as irreversible myocardial necrosis and decreased cardiac function.^[^ ^3^ ^]^ Currently, the efficacy of cardiac regenerative strategies for MI treatment still needs to be improved due to the poor self‐regeneration capability of CMs.^[^ ^3^ ^]^ Stem cell implantation and gene therapy utilize stem cells or genes to promote cardiac regeneration, which suffer from complex preparation, low cell survival and retention rate, and low gene transfection efficiency.^[^ ^4^ ^]^ In addition, the therapeutic effects including drug and cell therapies are greatly limited by the cardiac microenvironment, which might cause cardiac remodeling after MI and lead to heart failure.^[^ ^5–8^ ^]^ Therefore, myocardial repair strategies targeting the MI microenvironment such as reactive oxygen species (ROS), macrophages, and insufficient angiogenesis could be promising for MI treatment. In the early stage of MI, the level of ROS increases significantly due to the hypoxic microenvironment, which might cause irreversible damage to CMs and vascular cells.^[^ ^9^ ^]^ Moreover, ROS can further trigger severe inflammation by rapidly stimulating signal transduction to produce inflammatory cytokines.^[^ ^10^ ^]^ Meanwhile, macrophages secrete numerous inflammatory factors to direct an inflammatory response in the MI region.^[^ ^11^, ^12^ ^]^ Macrophages are the primary responder cells of the immune system that infiltrate the infarcted myocardium for cardiac repair and are broadly divided into proinflammatory phenotype (M1) and anti‐inflammatory phenotype (M2).^[^ ^13^, ^14^ ^]^ After MI occurs, M1 macrophages mainly remove debris, necrotic CMs, and apoptotic neutrophils, thereby triggering inflammation.^[^ ^15^, ^16^ ^]^ Then in the subsequent inflammation resolution stage, M2 macrophages dominate, which facilitates tissue reconstruction and regeneration.^[^ ^14^, ^15^, ^16^, ^17^ ^]^ During this process, the regulation of macrophage phenotype and inflammatory responses is essential for cardiac recovery.^[^ ^13^, ^14^, ^15^, ^18^ ^]^ Therefore, the combination of ROS scavenging to suppress the oxidative stress damage and macrophage polarization to anti‐inflammatory M2 phenotype in the MI microenvironment could be desirable for MI treatment.

Biomaterials such as graphene oxide,^[^ ^7^, ^19^, ^20^ ^]^ fullerenol,^[^ ^8^ ^]^ chitosan,^[^ ^6^ ^]^ poly(*β*‐amino esters),^[^ ^21^ ^]^ and polyurethane containing thioketal linkages^[^ ^22^ ^]^ have been exploited to promote myocardial repair by eliminating ROS. However, the regulation of ROS by these biomaterials may not be sufficient to meet the needs of MI treatment, so they usually work together with stem cell implantation,^[^ ^6^, ^7^, ^8^, ^19^ ^]^ gene delivery,^[^ ^20^ ^]^ or anti‐inflammatory agents loading.^[^ ^21^, ^22^ ^]^ In addition, small antioxidant molecules including glutathione and ascorbic acid were conjugated onto polymer chains to attenuate oxidative stress.^[^ ^23^, ^24^ ^]^ To modulate MI immune microenvironment, anti‐inflammatory plasmids including interleukin 4 (IL‐4) and interleukin 10 (IL‐10) with stem cells were also applied to propagate regenerative M2 macrophages.^[^ ^20^, ^25^ ^]^ In these works, the recovery of cardiac function relies on the cooperation of biomaterials and stem cells, drugs, or genes, which is complicated. Moreover, there are potential safety issues in the degradation, biocompatibility, and clearance of these materials. Therefore, it still remains great challenge to develop accessible, safe, and effective biomaterials, which could realize ROS scavenging and M2 macrophage progression simultaneously to facilitate myocardial repair.

Naturally occurring components extracted from living organisms are favorable for biomedical applications due to the minimized adverse effects and biosafety in vivo.^[^ ^26^ ^]^ Melanins are widely distributed biopolymers, which possess inherent biocompatibility and fascinating physicochemical characteristics. Well‐dispersed spherical melanin nanoparticles (MNPs) could be extracted from cuttlefish ink, which contain various amino acids and monosaccharides.^[^ ^27^, ^28^ ^]^ In addition to inherent biocompatibility and degradability, MNPs also possess intriguing photothermal properties, free radical scavenging ability, and strong chelating capability.^[^ ^27^, ^28^, ^29^, ^30^ ^]^ Natural MNPs demonstrate a variety of biomedical applications including UV irradiation protection, cataract symptoms relief,^[^ ^30^ ^]^ cancer theranostics,^[^ ^27^, ^29^, ^31^ ^]^ and anti‐infective therapy.^[^ ^32^ ^]^ In addition, natural MNPs were found to polarize tumor‐associated macrophages from M2 to antitumor M1‐like phenotype.^[^ ^28^ ^]^ Moreover, artificial melanin‐like nanoparticles with plentiful reductive functional groups have exhibited great potential in tissue repair. They have been successfully applied to treat ROS‐associated diseases such as ischemic stroke,^[^ ^33^ ^]^ periodontal disease,^[^ ^34^ ^]^ acute peritonitis and acute kidney/lung injury.^[^ ^35^, ^36^ ^]^ Artificial melanin‐like nanoparticles‐containing hydrogels also demonstrate excellent tissue adhesiveness and antioxidant properties in tissue repair.^[^ ^37^ ^]^ In addition, hydrogels with natural MNPs were employed for photothermal cancer therapy^[^ ^38^ ^]^ and for skin wound healing through regulating the ROS level.^[^ ^39^ ^]^ However, the application of natural MNPs in macrophage polarization and myocardial repair still remains to be explored. As a natural component extracted from ocean algae, anionic polysaccharide alginate is promising in myocardial repair and regeneration due to its biocompatibility, nonthrombogenic feature, and structural similarity with extracellular matrix.^[^ ^40^ ^]^ Injectable alginate hydrogels are also widely used in cardiac repair to provide mechanical support in MI area,^[^ ^7^, ^8^, ^40^, ^41^ ^]^ which have shown promising prospects in clinical trials.^[^ ^42^, ^43^ ^]^ Generally, injectable alginate hydrogels were usually applied for the delivery of stem cells and biomaterials to promote cardiac repair, which is costly and complicated. Therefore, we were inspired to fabricate natural MNPs/alginate hydrogels for improved MNP retention, structural support, and MI microenvironment‐targeted cardiac repair.

**Figure 1:**
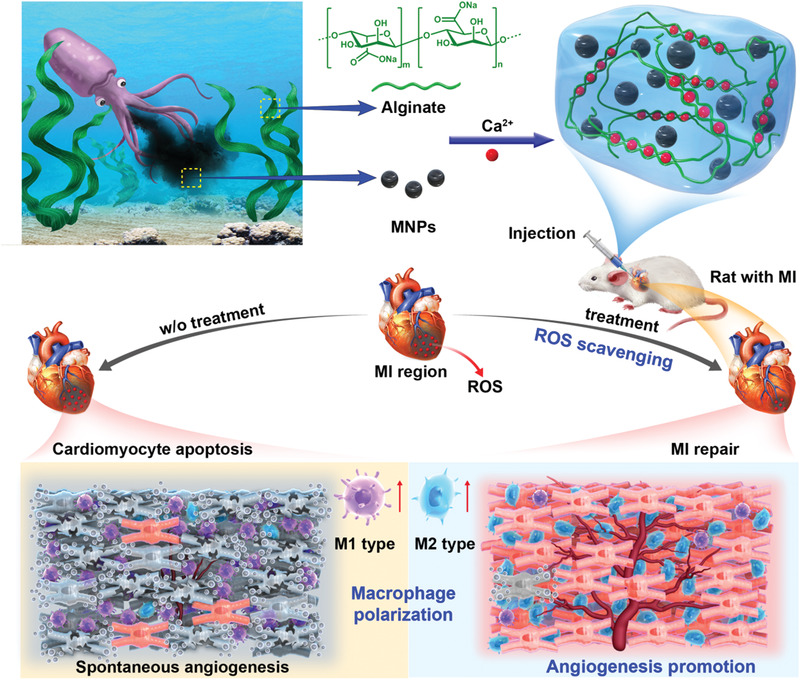
Schematic illustration of the preparation and therapeutic mechanism of MNPs/Alg hydrogel in cardiac repair in vivo.

Herein, we construct natural MNPs/alginate (MNPs/Alg) hydrogels through divalent cations (Ca^2+^) cross‐linking to regulate the oxidant stress and macrophage phenotype in the MI region, thereby facilitating myocardial repair (**Figure** **1**). Taking advantage of two marine‐derived natural biomaterials, MNPs/Alg hydrogels were expected to scavenge ROS and reprogram macrophages in the MI region. Survival capability of CMs could be greatly improved by the MNPs/Alg hydrogel through ROS scavenging. More interestingly, macrophage polarization toward regenerative M2 macrophages could be promoted in the MI microenvironment. To further evaluate the feasibility of the as‐prepared MNPs/Alg hydrogel in vivo, an MI rat model was employed. The antioxidant, anti‐inflammatory and proangiogenesis effects of the hydrogel were investigated in detail. The unique virtues of MNPs/Alg hydrogel and the potential value of natural biomaterials in cardiac repair were elucidated.

## 2 Results and Discussion

### 2.1 Preparation and Characterization of MNPs/Alg Hydrogels

**Figure 2:**
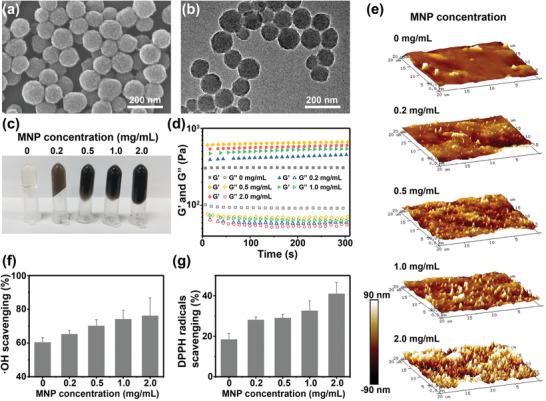
Preparation and characterization of MNPs/Alg hydrogels. a) SEM and b) TEM images of MNPs. c) Photographs of MNPs/Alg hydrogels with different MNP concentrations. d) Rheological analysis of MNPs/Alg hydrogels in a time sweep mode at 37 °C. Solid symbols stand for storage modulus G′ while hollow symbols stand for loss modulus G″. e) AFM images of surfaces of MNPs/Alg hydrogels with different MNP concentrations. Scavenging effect of MNPs/Alg hydrogels on f)·OH and g) DPPH radicals. (mean ± SD, *n* = 3.)

MNPs were first extracted from the ink sac of cuttlefish utilizing a facile centrifugation method.^[^ ^27^, ^28^ ^]^ As shown in the scanning electron microscope (SEM) and transmission electron microscope (TEM) images (**Figure** **2a**,b), MNPs displayed a spherical morphology with a diameter of 100–150 nm. The obtained MNPs were then added to the solution of sodium alginate. Calcium gluconate was employed to induce the cross‐linking of alginate chains. The gel structure is usually explained by the “egg box” cross‐linking model,^[^ ^44^, ^45^ ^]^ where Ca^2+^ interacts with guluronate blocks to link adjacent polymer chains. After calcium gluconate solution was introduced, MNPs/Alg hydrogels with different MNP concentrations could be achieved within 10 min, while MNPs were well dispersed to produce homogeneous hydrogels and the apparent color gradually darkened as the concentration of MNPs increased (Figure 2c). As the MNP concentration was increased from 0 to 2.0 mg mL^−1^, the rheology analysis of MNPs/Alg hydrogels was employed to evaluate the gelation kinetic (Figure 2d). It could be clearly found that the storage moduli (G′) of all hydrogels were higher than loss moduli (G″), indicating the successful formation of solid hydrogels. The G′ values ranged from 380 to 600 Pa, which could provide appropriate mechanical strength for cardiac repair.^[^ ^46^ ^]^ Generally speaking, scaffolds or patches used in myocardial repair demonstrate higher mechanical strength than that of the cardiac tissue,^[^ ^24^, ^47^ ^]^ which usually experience mechanical stress. However, mechanical strength is not the most important factor when injectable hydrogels are used to repair myocardium. The relatively soft hydrogel may facilitate the transmission of mechanical signal and coordination of the beat of the myocardium tissue, thereby promoting the rebuilding of cardiac function.^[^ ^48^ ^]^ As shown in the atomic force microscopy (AFM) images (Figure 2e), the pure alginate (Alg) hydrogel displayed a relatively uniform and flat surface topography, while the surface of MNPs/Alg hydrogels became rougher as the concentration of MNP increased. It was found that more cells adhered to the rough alginate hydrogel surfaces than to smooth surfaces.^[^ ^49^, ^50^ ^]^ The increase in surface roughness of MNPs/Alg hydrogels could promote the adhesion of CMs to hydrogels at the MI site and enhance repair effect.

To verify the hypothesis that MNPs endow hydrogels with the ability to scavenge ROS in infarcted cardiac tissues suffering from high oxidative stress, the antioxidant potential of MNPs/Alg hydrogels was investigated. As displayed in Figure 2f,g, the scavenging activity of MNPs/Alg hydrogels against hydroxyl radical (·OH) and 1′‐diphenyl‐2‐picrylhydrazyl radicals (DPPH) demonstrated an MNP concentration‐dependent manner, implying the important role of MNPs in radical scavenging. All MNPs/Alg hydrogels showed higher ·OH and DPPH scavenging capacity than the pure Alg hydrogel. When the concentration of MNPs is 1 mg mL^−1^, the scavenging effect on ·OH was ≈75%. The abundant antioxidant groups of MNPs such as phenolic hydroxyl were responsible for the scavenging of ROS.^[^ ^30^, ^36^ ^]^ The excellent antioxidant activity of MNPs/Alg hydrogels is beneficial to scavenge harmful ROS in the MI region and reshape the MI microenvironment.

### 2.2 Effect of MNPs/Alg Hydrogels on the Survival of CMs in ROS Microenvironment

**Figure 3:**
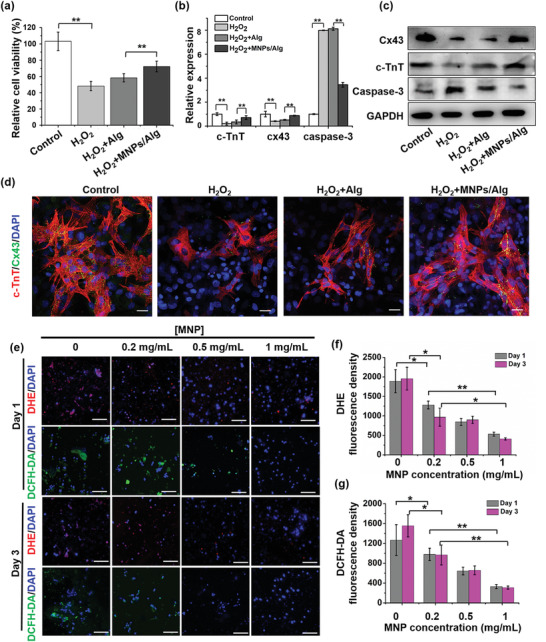
Effect of MNPs/Alg hydrogels on the survival of CMs in ROS Microenvironment. a) Viability of CMs incubated with Alg and MNPs/Alg hydrogels (concentration of MNPs: 1 mg mL^−1^) in medium containing 200 × 10^−6^ m H_2_O_2_ for 3 days. b) Gene expression of c‐TnT, cx43, and caspase‐3 in CMs analyzed by qRT‐PCR. c) Expression of c‐TnT, Cx43, and Caspase 3 in CMs by Western blotting on Day 3. d) Immunostaining of c‐TnT (red) and Cx43 (green) in CMs by confocal laser microscopy. e) Representative fluorescent images of intracellular superoxide anion radical activity (DHE) and total intracellular ROS (DCFH‐DA) treated with MNPs/Alg hydrogels on Day 1 and Day 3. Quantification of f) DHE and g) DCFH‐DA on Day 1 and Day 3. (mean ± SD, *n* = 3, *p < 0.05 and **p < 0.01, Student's *t* test.) Scale bar: 50 µm.

We first investigated the viability of neonatal rat CMs incubated with MNPs/Alg hydrogels. CMs were cultured with MNPs/Alg hydrogels with different MNP concentrations of 0, 0.2, 0.5, 1, and 2 mg mL^−1^, respectively. Alamar Blue assay was used to assess the viability of CMs after 3, 7, and 10 days, respectively (Figure S1, Supporting Information). MNPs/Alg hydrogels demonstrated good cytocompatibility during 3 or 7 days’ incubation. After 10 days, MNPs/Alg hydrogels containing 2 mg mL^−1^ MNPs exhibited an obvious inhibitory effect on the viability of CMs compared with hydrogels containing 1 mg mL^−1^ MNPs. Therefore, we finally chose MNPs/Alg hydrogels with 1 mg mL^−1^ MNPs for the subsequent experiments. For comparison, the viability of neonatal rat CMs mediated with free MNPs (0, 0.2, 0.5, 1, and 2 mg mL^−1^) was also studied. After 3 days’ incubation, an obvious inhibitory effect on the viability of CMs was observed when the concentration of MNPs was 2 mg mL^−1^ (Figure S2, Supporting Information). The Ca^2+^ cross‐linked alginate hydrogels are believed to be dissolved by the exchange reactions of Ca^2+^ with Na^+^ or other monovalent cations in the surrounding media.^[^ ^45^ ^]^ As shown in Figure S3 (Supporting Information), the MNPs/Alg hydrogel immersed in 37 °C culture medium was completely degraded within 21 days. According to the previous report, only isolated islands of alginate hydrogel were found in the MI area 4–6 weeks after injection.^[^ ^42^ ^]^ So it is speculated that the MNPs/Alg hydrogel played an important role in slow‐release of MNPs, which delayed the inhibitory effect on cell viability from 3 days to 10 days. To mimic the ROS microenvironment in vitro, 200 × 10^−6^ m H_2_O_2_ was employed to induce the oxidative stress, while the group of untreated CMs in normal culture medium was employed as the control group. The viability of CMs under ROS microenvironment was further assessed (**Figure** **3a**). H_2_O_2_ was observed to dramatically decrease the survival capacity of CMs due to oxidative stress injury. Compared with pure Alg hydrogel, MNPs/Alg hydrogel could significantly improve the viability of CMs under oxidant stress microenvironment. Therefore, it is speculated MNPs in MNPs/Alg hydrogel played a key role to scavenge ROS and reduce the oxidant stress injury of CMs.

To further investigate the effect on CMs, cellular expression of cardiac‐specific genes, proteins (cardiac troponin T (c‐TnT), connexin 43 (cx43)) and apoptosis‐related gene caspase‐3 in CMs was evaluated using quantitative real‐time polymerase chain reaction (qRT‐PCR) and Western blot analysis (Figure 3b,c). MNPs/Alg hydrogel was found to effectively upregulate the expression of cardiac‐specific contractile protein c‐TnT and gap junction protein Cx43 in CMs under the ROS microenvironment, which play an important role in myocardial repair.^[^ ^51^ ^]^ Meanwhile, the expression of apoptosis‐related Caspase‐3 was evidently inhibited, indicating that MNPs/Alg hydrogel could effectively prevent the apoptosis of CMs. Immunofluorescence images in Figure 3d further visualized the expression of c‐TnT and Cx43 in CMs. Few c‐TnT or Cx43 were expressed under the ROS microenvironment in the absence or presence of Alg hydrogel. In contrast, CMs treated with MNPs/Alg hydrogel exhibited much stronger c‐TnT and Cx43 signals which was similar to normal CMs. The above results confirmed that MNPs/Alg hydrogels could effectively protect CMs from oxidative stress damage, produce enhanced expression of Cx43, and facilitate cardiac repair.

To verify the ROS scavenging activity of MNPs/Alg hydrogels, dihydroethidium (DHE) and 2′,7′‐dichlorofluorescin diacetate (DCFH‐DA) staining was used to assess intracellular ROS levels, including superoxide anions and hydroxyl radicals. After 1 day, strong red fluorescence from DHE and green fluorescence from DCFH‐DA appeared around the nuclei (blue fluorescence from 4′,6′‐diamidino‐2‐phenylindole (DAPI)) in pure Alg hydrogel (Figure 3e), indicating a high level of ROS. With the increase of the MNP concentration in MNPs/Alg hydrogels, the intensity of fluorescence from both DHE and DCFH‐DA decreased. When the MNP concentration in MNPs/Alg hydrogel is 1 mg mL^−1^, few DHE or DCFH‐DA fluorescence could be observed. After 3 days, negligible fluorescence signals were found when the MNP concentration is 1 mg mL^−1^. Quantitative analysis of the fluorescence density demonstrated that the addition of MNPs in MNPs/Alg hydrogels significantly reduced the ROS levels in CMs, and the fluorescence density decreased with the increase of MNP concentration (Figure 3f,g). Taken together, MNPs/Alg hydrogels could improve the survival capability of CMs through ROS scavenging.

### 2.3 Effect of MNPs/Alg Hydrogel on Polarization of Macrophages and Angiogenesis

Oxidative stress and inflammation activation in the initial stage of MI is the key mechanisms of ischemic myocardial injury. As the main immune cells that infiltrate the infarcted myocardium, macrophages are rapidly activated, leading to the secretion of harmful inflammatory cytokines and more severe myocardial injury.^[^ ^52^ ^]^ M1 macrophages are closely related to inflammation, while M2 macrophages play an important role in facilitating cardiac repair. Inspired by the report that natural MNPs could reprogram tumor‐associated macrophages,^[^ ^28^ ^]^ we studied the effect of MNPs/Alg hydrogel on the polarization of macrophages in ROS microenvironment that simulates MI region in vitro. First, mouse bone marrow‐derived mononuclear macrophages (BMDMs) were pretreated with lipopolysaccharide (LPS), followed by the addition of H_2_O_2_ to promote M1 differentiation in the ROS microenvironment. As shown in Figure S4 (Supporting Information), the expression of the proinflammatory inducible nitric oxide synthase (iNOS), C–C motif chemokine ligand 2 (CCL‐2), and tumor necrosis factor alpha (TNF‐*α*) genes were significantly upregulated in LPS‐stimulated BMDMs. At the same time, the expression of anti‐inflammatory genes related with reparative M2 macrophages (IL‐10, transforming growth factor *β* (TGF‐*β*), and Arginase1 (Arg1)) did not change much. After BMDMs were cultured with pure Alg or MNPs/Alg hydrogels for 24 h, the expression of proinflammatory (TNF‐*α*, CCL‐2, and iNOS) and anti‐inflammatory genes (IL‐10, TGF‐*β*, and Arg1) were further evaluated by qRT‐PCR. Interestingly, the expressions of proinflammatory genes were significantly downregulated by MNPs/Alg hydrogel compared with pure Alg hydrogel (Figure S4, Supporting Information), demonstrating the anti‐inflammatory effect of MNPs. It was also observed that neither Alg nor MNPs/Alg hydrogel could significantly upregulate the expression of anti‐inflammatory genes in LPS‐stimulated BMDMs. Therefore, MNPs/Alg hydrogel could effectively reduce the inflammatory response under the ROS environment but no obvious polarization of macrophages from LPS‐stimulated M1 to M2 macrophages was observed.

**Figure 4:**
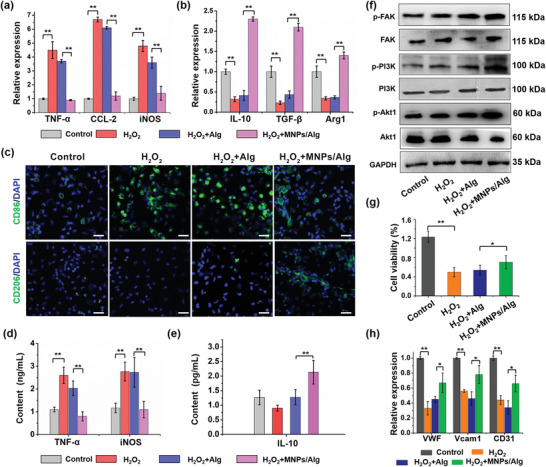
Effect of MNPs/Alg hydrogel on polarization of macrophages and angiogenesis in the ROS microenvironment. qRT‐PCR assay of the expression of a) proinflammatory genes (TNF‐*α*, CCL‐2, iNOS) and b) anti‐inflammatory genes (IL‐10, TGF‐*β*, and Arg1) in BMDMs treated with Alg or MNPs/Alg hydrogels for 1 day. c) Representative immunofluorescence images of CD86 and CD206 with different treatments in BMDMs for 1 day. Scale bar: 50 µm. d,e) Quantification analysis for proinflammatory (TNF‐*α* and iNOS) and anti‐inflammatory cytokines (IL‐10) secretion in BMDMs for 1 day. f) Western blot analysis of the levels of FAK, PI3K, and Akt1 phosphorylation in BMDMs treated with Alg or MNPs/Alg hydrogels for 1 day. g) Alamar Blue assay of HUVEC treated with Alg or MNPs/Alg hydrogels for 3 days. h) qRT‐PCR assay of gene expression of angiogenesis‐related genes (VWF, Vcam1, and CD31) in HUVEC treated with Alg or MNPs/Alg hydrogels for 3 days. (mean ± SD, *n* = 3, *p < 0.05 and **p < 0.01, Student's *t* test.)

We then investigated the effect of MNPs/Alg hydrogel on the polarization of BMDMs under the ROS microenvironment. Primary BMDMs were isolated and cultured for 8 days, followed by the addition of H_2_O_2_ and Alg or MNPs/Alg hydrogels for 24 h. Afterward, the cells were collected and analyzed by qRT‐PCR, immunofluorescence imaging, and enzyme‐linked immunosorbent assay (ELISA). As demonstrated in **Figure** **4a**,b, MNPs/Alg hydrogel significantly downregulated the expressions of proinflammatory genes (TNF‐*α*, CCL‐2, and iNOS) and evidently upregulated the expression of genes related with reparative M2 macrophages (IL‐10, TGF‐*β*, and Arg‐1), respectively. Immunofluorescence staining was subsequently performed to show the expression of CD86 (M1 macrophage surface biomarker) and CD206 (M2 macrophage surface biomarker) of BMDMs after different treatments (Figure 4c). The amount of CD86‐positive BMDMs increased significantly after H_2_O_2_ was added, indicating the polarization of BMDMs to M1 phenotype. Meanwhile, there was no significant change in the amount of CD206‐positive cells. Obviously, MNPs/Alg hydrogel decreased the signal of CD86 and increased the signal of CD206, implying MNPs/Alg hydrogel‐induced polarization from M1 to M2 macrophages. Furthermore, BMDMs treated with MNPs/Alg hydrogel displayed obvious morphological changes with an elongated shape of M2 phenotype while other groups exhibited a round and flattened morphology for BMDMs and M1 counterparts^[^ ^20^, ^28^ ^]^ (Figure S5, Supporting Information). In addition, ELISA demonstrated a significant reduction in proinflammatory cytokines (TNF‐*α* and iNOS, Figure 4d) and a substantial increase of M2‐associated anti‐inflammatory cytokine IL‐10 (Figure 4e) in BMDMs treated with MNPs/Alg hydrogels. All these results confirmed that in the ROS microenvironment, MNPs/Alg hydrogel could inhibit the differentiation of BMDMs to M1 phenotype and promote the polarization to M2 macrophages. It was reported that MNPs could polarize tumor‐associated macrophages from M2 to antitumor M1‐like phenotype through internalization and accumulation in lysosomes of macrophages.^[^ ^28^ ^]^ However, MNPs trapped in MNPs/Alg hydrogel might not be easily internalized by macrophages. We further studied the effect of free MNPs on the polarization of BMDMs. Immunofluorescence staining and qRT‐PCR results (Figure S6, Supporting Information) demonstrated that the expression of M1 macrophage surface biomarker (CD86) and proinflammatory genes (TNF‐*α*, CCL‐2, and iNOS) were upregulated, indicating the polarization of BMDMs to M1 phenotype. After MNPs were encapsulated in the hydrogel, the expressions of CD86 and proinflammatory genes were significantly downregulated by the MNPs/Alg hydrogel, implying the inhibition in M1 differentiation. Then MNPs and the MNPs/Alg hydrogel were incubated with BMDMs pretreated by LPS (M1 phenotype), respectively. As exhibited in Figure S7 (Supporting Information), compared with MNPs, the MNPs/Alg hydrogel could effectively downregulate the expression of M1 macrophage‐related genes and CD86, while upregulating the expression of M2 macrophage‐related genes and CD206. These results verify our hypothesis that the encapsulation of MNPs promotes the polarization of M2 macrophages. Herein, MNPs/Alg hydrogel was proved to induce M2 macrophage polarization by regulating the H_2_O_2_‐induced ROS microenvironment.

Since the macrophage polarization toward reparative M2 macrophages is important for myocardial regeneration, the mechanism behind needs to be elucidated. It was speculated that MNPs could react with ROS to produce O_2_.^[^ ^33^ ^]^ Notably, the simultaneous O_2_ generation and ROS scavenging were found to successfully drive polarization of M1 to M2 mancrophages under hypoxic and inflammatory conditions.^[^ ^53^ ^]^ In order to exploit the molecular mechanism of the MNPs/Alg hydrogel‐induced M2 macrophage polarization in the ROS microenvironment, the protein kinase B (Akt) signaling pathway was further studied.^[^ ^54^, ^55^ ^]^ The phosphoinositide 3‐kinase (PI3k)/Akt pathway was considered to regulate macrophage survival, migration, and proliferation, which plays an important role in macrophage activation.^[^ ^54^ ^]^ Western blot was used to analyze the phosphorylation levels of focal adhesion kinase (FAK), PI3K, and Akt1 after BMDMs were cultured with pure Alg or MNPs/Alg hydrogels under the ROS microenvironment. As shown in Figure 4f, the MNPs/Alg hydrogel was found to increase the phosphorylation levels of FAK, PI3K, and Akt1, which is consistent with the previously reported results.^[^ ^54^ ^]^ Subsequently, BEZ235 (200 ng mL^−1^), an inhibitor that blocks the downstream signals of PI3K/Akt1/mammalian target of rapamycin (mTOR) pathway, was introduced to the MNPs/Alg hydrogel group. An obvious inhibitory effect on the macrophage polarization was observed and the expression of M2 macrophage‐related genes (IL‐10, TGF‐*β*, and Arg1) decreased obviously after being incubated with BEZ235 (Figure S8, Supporting Information). These results suggest that BEZ235 reduced the polarization level of BMDMs treated with the MNPs/Alg hydrogel. Therefore, PI3K/Akt1/mTOR was considered as the main pathway for MNPs/Alg hydrogel‐induced M2 macrophage polarization.

Then we explored the effect of MNPs/Alg hydrogel on angiogenesis, which plays an important role in preserving cardiac functions during MI.^[^ ^2^ ^]^ The cell viability of human umbilical vein endothelial cells (HUVECs) in the ROS microenvironment was first assessed by Alamar Blue assay. After 3 days of continuous culture, the viability of HUVECs was significantly inhibited due to the oxidative stress injury (Figure 4g), while the MNPs/Alg hydrogel increased the viability of endothelial cells to a certain extent. Subsequently, qRT‐PCR was utilized to detect the expression of angiogenesis‐related genes including von Willebrand factor (VWF), vascular cell adhesion molecule 1 (Vcam1) and CD31. As indicated in Figure 4h, the MNPs/Alg hydrogel remarkably upregulated the expressions of angiogenesis‐related genes compared with pure Alg hydrogel. Similar to the situation of CMs, MNPs/Alg hydrogel could improve the survival capability of HUVECs through ROS scavenging to facilitate angiogenesis in the MI region. Taken together, MNPs/Alg hydrogels could effectively drive the polarization of macrophages to M2 phenotype and improve angiogenesis in the ROS microenvironment.

### 2.4 Antiapoptosis and ROS Scavenging Effect of MNPs/Alg Hydrogel In Vivo

**Figure 5:**
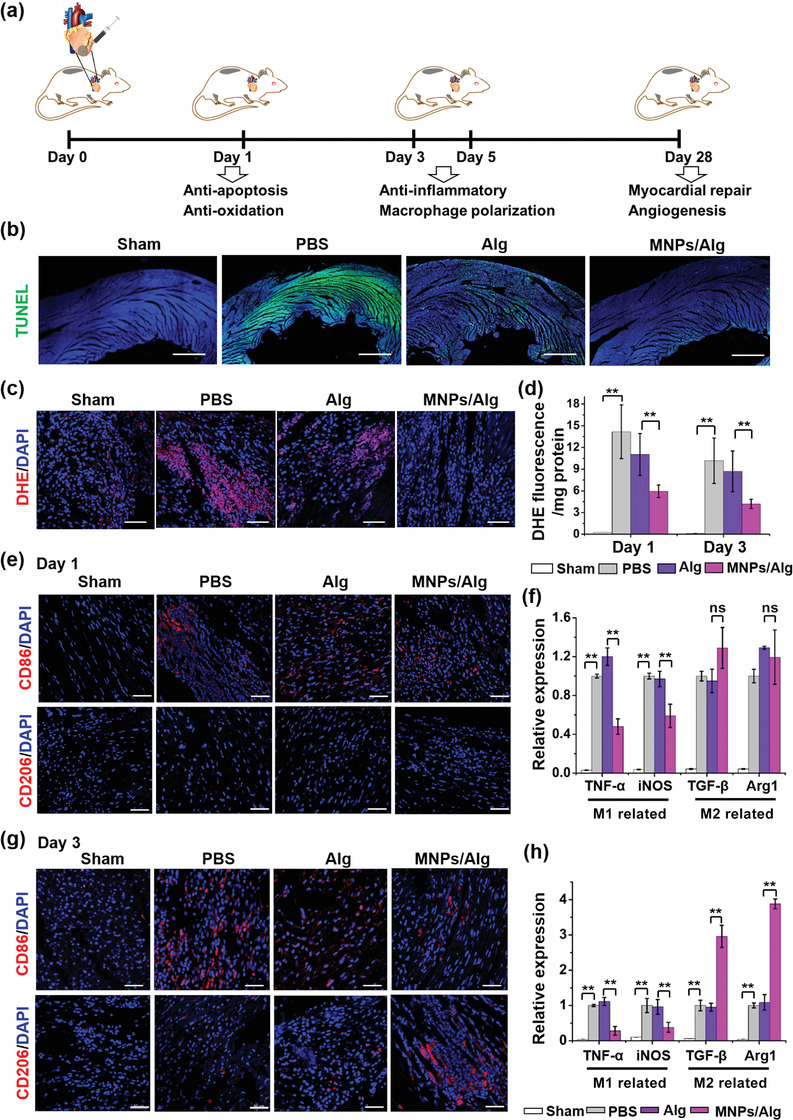
Effect of MNPs/Alg hydrogel on the ROS and immune microenvironment in vivo in the early stages. a) Time axis of the myocardial repair process we detected in the MI region with MNPs/Alg hydrogel. b) Fluorescent images of the apoptotic cells at the infarct zone 1 day after injection by TUNEL‐positive expression (green). c) Representative fluorescent images of superoxide anion radical activity (DHE, red) of heart sections 1 day after injection. d) Quantification of DHE by ROS detection kit (DHE) after treatment on Day 1 and Day 3. e) Immunofluorescence images of CD86 and CD206 in the MI region on Day 1 after treatments. f) qRT‐PCR assay of the expression of M1‐ (TNF‐*α* and iNOS) and M2‐related (TGF‐*β* and Arg1) genes in MI region on Day 1. g) Immunofluorescence images of CD86 and CD206 in the MI region 3 days after treatments. h) qRT‐PCR assay of the gene expression of M1 and M2 marker on Day 3. (mean ± SD, *n* = 3, *p < 0.05, **p < 0.01, ^ns^ *p* > 0.05, Student's *t* test.) Scale bar: 50 µm.

Encourage by the promising in vitro results, we carried out the in vivo experiments employing the MI model of rats. The in vivo degradation of the Alg hydrogel was first monitored through subcutaneous implantation since the rate of degradation has a direct effect on the interaction between MNPs and the body. The degradation rate was calculated by the weight change of the hydrogel left in the subcutaneous site. As demonstrated in Figure S9a (Supporting Information), slight weight loss (≈8%) after 7 days and ≈20% weight loss after 14 days was observed. The slow degradation behavior of Alg hydrogel is consistent with the previous report,^[^ ^56^ ^]^ which is beneficial to provide mechanical support and improve MNP retention in the MI region. In addition, the introduction of MNPs did not change the degradation rate of the hydrogel (Figure S9b,c, Supporting Information). To investigate the antiapoptosis effect of MNPs/Alg hydrogel, the sham group (underwent thoracotomy only without ligation of left anterior descending artery) was applied as the positive control, while the MI model injected with PBS was employed as the negative control. MI induces severe apoptosis of CMs and it has been reported that numerous CM apoptosis were observed in the infarcted areas 1 day after MI.^[^ ^57^ ^]^ At the same time, inflammatory M1 macrophages dominate on Days 1–3 post‐MI, which differentiate to reparative M2 phenotype between 3 and 5 days after MI to regulate angiogenesis and prepare for tissue regeneration.^[^ ^14^, ^58^ ^]^ Therefore, we investigated the MI region of hearts at different stages after the direct injection of MNPs/Alg hydrogel in vivo (**Figure** **5a**). First, the CM apoptosis of MI was evaluated by terminal deoxynucleotidyl transferase (TdT) dUTP nick‐end labeling (TUNEL) assay 1 day after the treatment. As shown in Figure 5b, the apoptosis of CMs was obviously reduced in the heart sections treated with MNPs/Alg hydrogel compared with the pure Alg hydrogel and PBS groups, suggesting that the apoptosis of CMs was effectively inhibited 1 day after the injection of MNPs/Alg hydrogel. To verify the hypothesis that the MNPs/Alg hydrogel improved the survival of CMs through modulating the ROS microenvironment, DHE staining was used to visualize the intracellular superoxide anions. As shown in Figure 5c,d, obvious DHE signal was detected 1 day after the injection of PBS, indicating the existence of a large amount of ROS accumulated in MI region. Meanwhile, DHE fluorescence was observed to decrease significantly after the injection of MNPs/Alg hydrogel compared with the pure Alg hydrogel group on Day 1 and Day 3. These results confirm the antioxidant property of MNPs and MNPs/Alg hydrogel, which could protect CMs from apoptosis by eliminating ROS in vivo in the early stage of MI.

### 2.5 Macrophages Polarization to Reparative M2 Phenotype after Injection of MNPs/Alg Hydrogel In Vivo

To evaluate the polarization of macrophages, immunofluorescence staining and qRT‐PCR of heart tissues after different treatments were performed on Day 1, Day 3, and Day 5, respectively after MI. The expressions of surface biomarkers and inflammation‐related genes were detected to analyze the proinflammatory and anti‐inflammatory phase. Immunofluorescence images in Figure 5e demonstrated that 1 day after treatment, the expression of the CD86 (M1 macrophages surface marker) in the MI region of PBS and Alg hydrogel groups was obvious. Interestingly, the amount of CD86‐positive M1 macrophages in the MNPs/Alg hydrogel group was significantly reduced compared with PBS and Alg hydrogel groups. By contrast, almost no CD206‐positive M2 macrophages were observed on the first day. Simultaneously, the inflammatory cytokine TNF‐*α* was evaluated by immunofluorescence staining, suggesting the upregulated expression of TNF‐*α* in the PBS and Alg hydrogel groups (Figure S10, Supporting Information). In comparison, TNF‐*α* expression decreased to a certain extent after the injection of MNPs/Alg hydrogel. qRT‐PCR analysis verified that the downregulation of inflammatory TNF‐*α* and iNOS expression (Figure 5f). There was no significant difference in the expression of anti‐inflammatory cytokines TGF‐*β* and Arg1 among different treatment groups. These results indicate that in the early stage of MI (1 day), the introduction of MNPs/Alg hydrogel could downregulate inflammatory genes and M1 macrophages while M2 macrophages did not show noticeable changes.

Interestingly, the expression of M2 macrophage markers showed obvious differences 3 days after treatments. Immunofluorescence images in Figure 5g show a significant upregulation of CD206 expression in the MNPs/Alg hydrogel group compared with PBS and Alg hydrogel groups, suggesting that MNPs/Alg hydrogel induced M2 macrophages polarization in the MI region. Simultaneously, CD86‐positive cells were also substantially reduced in the MNPs/Alg hydrogel group, demonstrating that MNPs/Alg hydrogel downregulated M1 macrophages to inhibit the inflammatory response of early MI. As displayed in Figure 5h, qRT‐PCR analysis confirmed the significant inhibitory effect of MNPs/Alg hydrogel on the expression of inflammatory cytokines TNF‐*α* and iNOS. In addition, the anti‐inflammatory cytokines TGF‐*β* and Arg1 expressions were also significantly upregulated on Day 3. These findings are consistent with the immunofluorescence staining results (Figure S11, Supporting Information).

Next, the heart tissues with different treatments were tracked and analyzed after 5 days’ treatment. The immunofluorescence images of M1 markers (CD86) and M2 markers (CD206 and Arg1) confirmed the propagated macrophage polarization from M1 to M2 phenotype 5 days after the injection of MNPs/Alg (Figure S12a, Supporting Information). As demonstrated in Figure S12b,c (Supporting Information), the expression of proinflammatory genes TNF‐*α*, CCL‐2, and iNOS in PBS and Alg hydrogel groups still remained high levels on Day 5, which were significantly downregulated in the MNPs/Alg hydrogel group. The expression of M2 phenotype related TGF‐*β*, IL‐10, and Arg1 were significantly higher in the MNPs/Alg hydrogel group than other groups, which is consistent with the results on Day 3. Taken together, MNPs/Alg hydrogel was proved to downregulate proinflammatory M1 macrophages at the MI region 1–3 days after treatment and induce macrophage polarization to M2 phenotype on days 3–5 for better myocardial repair.

### 2.6 Therapeutic Effects of MNPs/Alg Hydrogel in MI Rat Model

**Figure 6:**
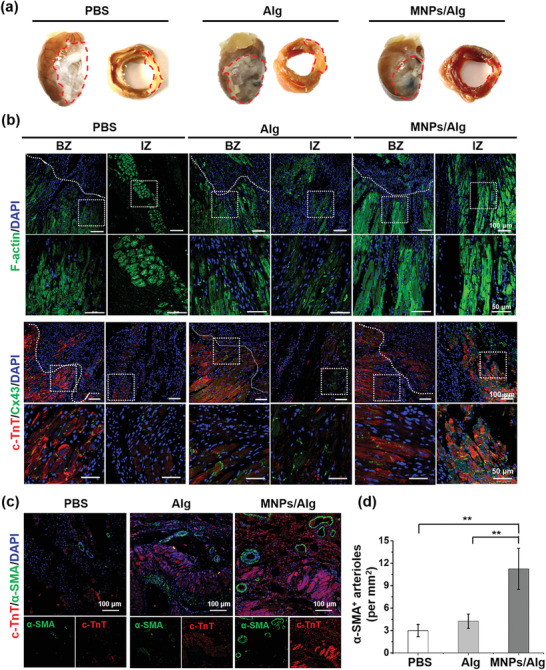
Effects of MNPs/Alg hydrogels on the morphology of infarcted hearts, cytoskeletal structure, gap junction, and angiogenesis 28 days after different treatments. a) Photographs of hearts and TTC‐stained heart sections after different treatments for 28 days (infarct areas separated with dots). b) Representative immunofluorescent images of F‐actin (green), c‐TnT (red), and Cx43 (green) in border zone (BZ) and infarct zone (IZ) of different groups 28 days after MI (DAPI in blue). Lower panels show higher magnification images of the regions marked by white boxes in the corresponding upper panels. c) Immunohistochemical staining of c‐TnT (red) and *α*‐SMA (green) in the MI region 28 days after treatment. d) Quantification analysis of *α*‐SMA‐positive arterioles. (mean ± SD, *n* = 5, *p < 0.05 and **p < 0.01, Student's *t* test.)

The therapeutic effect of MNPs/Alg hydrogel on myocardial repair in vivo was then assessed after 28 days of treatment. The rats were sacrificed and heart samples in different groups were collected to evaluate the morphology of hearts, cytoskeletal structure, gap junction, and angiogenesis. As shown in **Figure** **6a**, the size of the heart after MI was observed to increase compared with the normal heart (Figure S13, Supporting Information). After the treatment with Alg or MNPs/Alg hydrogels, the size of hearts reduced and the heart in MNPs/Alg hydrogel group almost returned to normal. The infarct area was further distinguished by the TTC‐staining of heart sections. The heart treated with MNPs/Alg hydrogel demonstrated much smaller infarct area compared with PBS or Alg hydrogel group (Figure 6a). In addition, heart sections after 28 days’ treatment were studied by immunofluorescence staining to further assess the cytoskeletal structures in the MI region (Figure 6b). It was observed that the striated structure and F‐actin‐positive area in MNPs/Alg hydrogel group were much clearer than those in PBS or pure Alg hydrogel group. Moreover, markers of mature CMs in heart sections (c‐TnT and Cx43) were upregulated obviously compared with those in PBS or Alg hydrogel group. High level of Cx43 expression in the MNPs/Alg hydrogel group indicates the remodeling and formation of gap junctions between CMs, which is crucial in cardiac repair.^[^ ^20^ ^]^ The excellent myocardial repair function of MNPs/Alg hydrogel might be attributed to the protection of CMs in the ROS microenvironment at the initial stage of MI, and the subsequent formation of gap junctions between CMs by regulation of macrophages, thereby facilitating cardiac repair.

Angiogenesis is critical for myocardial repair after MI to recover oxygen and rescue CMs.^[^ ^2^ ^]^ Therefore, we further studied the effect of MNPs/Alg hydrogel on the vascular density in the MI region (Figure 6c,d). The blood vessel density was evaluated by immunofluorescence staining of *α*‐smooth muscle actin (*α*‐SMA), which is a marker of mature neovessels.^[^ ^22^ ^]^ Compared with PBS group, more neovessels were found in Alg hydrogel group. More importantly, the highest arteriole density was observed in the MNPs/Alg hydrogel group (Figure 6c). Quantification analysis reveals the significant higher number of arterioles in the MNPs/Alg hydrogel group than other groups (Figure 6d). These results suggest that the therapeutic effect of MNPs/Alg hydrogel on left ventricular reconstruction may be attributed to the increased vessel density in the infract region. Therefore, MNPs/Alg hydrogel plays a combined role in regulating the inflammatory MI microenvironment and promoting angiogenesis, thereby effectively reducing adverse cardiac remodeling.

**Figure 7:**
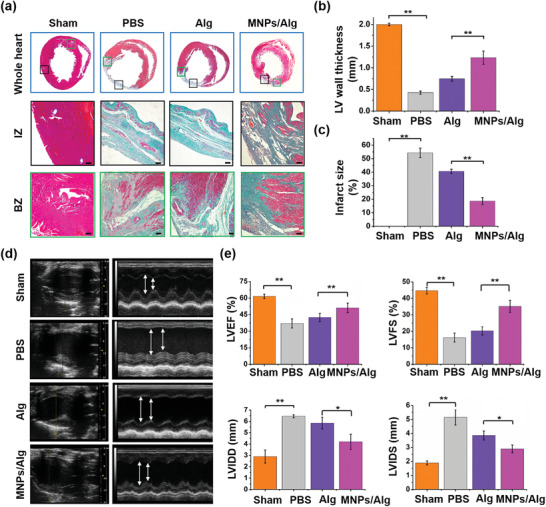
Effect of MNPs/Alg hydrogel on cardiac structure of infarcted hearts and recovery of cardiac function after 28 days’ treatment. a) Masson's trichrome staining images of border zone (BZ) and infarct zone (IZ) of infarcted hearts after different treatments for 28 days. Scale bar: 100 µm. Quantitative analysis of b) infarct size and c) infarct wall thickness. d) Echocardiographic images of MI rats in PBS, Alg hydrogel and MNPs/Alg hydrogel groups, and e) related LVEF, LVFS, LVIDD, and LVIDS analysis. (mean ± SD, *n* = 10, *p < 0.05 and **p < 0.01, Student's *t* test.)

Masson's trichrome staining was then performed to analyze the cardiac fibrosis and collagen deposition after 28 days’ treatment. As shown in **Figure** **7a**, compared with PBS group, the injection of pure Alg hydrogel could restore the infracted heart to a certain extent. To be noticed, the least fibrosis (blue fibroblasts and collagen) could be observed at both the infarct and border zone in the MNPs/Alg hydrogel group, demonstrating the smallest lesion areas. In particular, the thickness of the left ventricular (LV) wall in the MNPs/Alg hydrogel group increased significantly compared with PBS and Alg hydrogel groups (from 0.42 ± 0.035 to 1.23 ± 0.15 mm, Figure 7b). The infarct size in MNPs/Alg hydrogel group decreased substantially from 54.3% to 20.7% accordingly and most fibrotic tissues were recovered to the normal red myocardium (Figure 7c).

Finally, cardiac functions were evaluated 28 days after MI by echocardiography (Figure 7d). Compared with normal hearts, the infarcted hearts treated with the PBS exhibited typical MI characteristics with a significantly reduced left ventricular ejection fraction (LVEF) and left ventricular fraction shortening (LVFS), as well as substantially increased left ventricular internal diameter at the end systole (LVIDS) and left ventricular internal diameter at the end diastole (LVIDD). Pure Alg hydrogel demonstrated limited effect on cardiac repair with increased LVEF and decreased LVIDS. LVEF was improved by ≈8% by the Alg hydrogel, which is comparable to the previous reports.^[^ ^42^ ^]^ Notably, a significant increase in LVEF and LVFS, as well as a substantial decrease in LVIDS and LVIDD was found in the MNPs/Alg hydrogel group, suggesting the enhanced pumping function and ventricular filling. It was found that the MNPs/Alg hydrogel increased LVEF by ≈20%, verifying the significantly improved performance in myocardial repair. Compared with other systems targeting the MI microenvironment that increase the performance by ≈15–20%,^[^ ^5^, ^59^, ^60^ ^]^ the injectable natural MNPs/Alg hydrogel developed in this work could achieve improved cardiac function without the assistance of stem cells, drugs, or genes. The significantly improved restoration of cardiac function further confirms the advantages and application prospects of MNPs/Alg hydrogel in cardiac repair.

## 3 Conclusion

In summary, novel injectable natural MNPs/Alg hydrogels with adjustable MNP concentrations were successfully achieved for cardiac repair by modulating the MI microenvironment in the absence of stem cells, drugs, or genes. With the dual roles of MNP in antioxidation and macrophage polarization, the MNPs/Alg hydrogel could attenuate inflammation by scavenging harmful ROS and promoting macrophage polarization to regenerative M2 phenotype. In addition, injectable alginate hydrogel could provide mechanical support and improve MNP retention in the MI region. As a result, the MNPs/Alg hydrogel could protect CMs from oxidant stress injury and induce M2 macrophage polarization through the PI3k/Akt1/mTOR signaling pathway. After the injection of MNPs/Alg hydrogel in vivo, the survival of CMs was significantly improved by regulating the ROS microenvironment. Notably, polarization of macrophages toward M2 phenotype was observed for better myocardial repair. Therefore, promoted formation of gap junctions between CMs and improved angiogenesis was readily induced by the MNPs/Alg hydrogel, effectively reducing adverse cardiac remodeling. The corresponding infarct size was reduced and the thickness of the left ventricular wall was increased substantially. The significantly improved recovery of cardiac function further verified the excellent therapeutic efficacy of MNPs/Alg hydrogel in cardiac repair. The present work introduces the MNPs/Alg hydrogel composed of two marine‐derived natural biomaterials in regulating ROS and immune MI microenvironment for cardiac repair.

## 4 Experimental Section

Details on the synthesis and characterizations of MNPs/Alg hydrogels, procedures of ROS scavenging, isolation of cardiomyocytes, bone marrow‐derived macrophages, viability of cardiomyocytes, assessment of macrophage polarization, in vivo study in myocardial infarction rat model, statistical analysis, and any associated references are given in the Supporting Information.

## Conflict of Interest

The authors declare no conflict of interest.

## Supporting information

Supporting Information

## Data Availability

Research data are not shared.

## References

[1] C. L. Hastings , E. T. Roche , E. Ruiz‐Hernandez , K. Schenke‐Layland , C. J. Walsh , G. P. Duffy , Adv. Drug Delivery Rev. 2015, 84, 85. 10.1016/j.addr.2014.08.006 25172834

[2] J. J. Nie , B. Qiao , S. Duan , C. Xu , B. Chen , W. Hao , B. Yu , Y. Li , J. Du , F. J. Xu , Adv. Mater. 2018, 30, 1801570. 10.1002/adma.201801570 29920798

[3] D. Lloyd‐Jones , R. J. Adams , T. M. Brown , M. Carnethon , S. Dai , G. De Simone , T. B. Ferguson , E. Ford , K. Furie , C. Gillespie , A. Go , K. Greenlund , N. Haase , S. Hailpern , P. M. Ho , V. Howard , B. Kissela , S. Kittner , D. Lackland , L. Lisabeth , A. Marelli , M. M. McDermott , J. Meigs , D. Mozaffarian , M. Mussolino , G. Nichol , V. L. Roger , W. Rosamond , R. Sacco , P. Sorlie , R. Stafford , T. Thom , S. Wasserthiel‐Smoller , N. D. Wong , J. Wylie‐Rosett , Circulation 2010, 121, 948. 20177011 10.1161/CIRCULATIONAHA.109.192666

[4] M. A. Borrelli , H. R. Turnquist , S. R. Little , Adv. Drug Delivery Rev. 2021, 173, 181. 10.1016/j.addr.2021.03.014 PMC8178247 33775706

[5] Y. S. Kim , H. Y. Jeong , A. R. Kim , W. H. Kim , H. Cho , J. Um , Y. Seo , W. S. Kang , S. W. Jin , M. C. Kim , Y. C. Kim , D. W. Jung , D. R. Williams , Y. Ahn , Sci. Rep. 2016, 6, 30726. 27510556 10.1038/srep30726 PMC4980696

[6] Z. Liu , H. Wang , Y. Wang , Q. Lin , A. Yao , F. Cao , D. Li , J. Zhou , C. Duan , Z. Du , Y. Wang , C. Wang , Biomaterials 2012, 33, 3093. 22265788 10.1016/j.biomaterials.2011.12.044

[7] G. Choe , S. W. Kim , J. Park , J. Park , S. Kim , Y. S. Kim , Y. Ahn , D. W. Jung , D. R. Williams , J. Y. Lee , Biomaterials 2019, 225, 119513. 31569016 10.1016/j.biomaterials.2019.119513

[8] T. Hao , J. Li , F. Yao , D. Dong , Y. Wang , B. Yang , C. Wang , ACS Nano 2017, 11, 5474. 28590722 10.1021/acsnano.7b00221

[9] H. Tsutsui , S. Kinugawa , S. Matsushima , Am. J. Physiol.: Heart Circ. Physiol. 2011, 301, H2181. 21949114 10.1152/ajpheart.00554.2011

[10] M. Hori , K. Nishida , Cardiovasc. Res. 2009, 81, 457. 19047340 10.1093/cvr/cvn335

[11] J. S. Burchfield , M. Xie , J. A. Hill , Circulation 2013, 128, 388. 23877061 10.1161/CIRCULATIONAHA.113.001878 PMC3801217

[12] S. A. Dick , J. A. Macklin , S. Nejat , A. Momen , X. Clemente‐Casares , M. G. Althagafi , J. Chen , C. Kantores , S. Hosseinzadeh , L. Aronoff , A. Wong , R. Zaman , I. Barbu , R. Besla , K. J. Lavine , B. Razani , F. Ginhoux , M. Husain , M. I. Cybulsky , C. S. Robbins , S. Epelman , Nat. Immunol. 2019, 20, 29. 30538339 10.1038/s41590-018-0272-2 PMC6565365

[13] J. M. Lambert , E. F. Lopez , M. L. Lindsey , Int. J. Cardiol. 2008, 130, 147. 18656272 10.1016/j.ijcard.2008.04.059 PMC2857604

[14] S. Frantz , M. Nahrendorf , Cardiovasc. Res. 2014, 102, 240. 24501331 10.1093/cvr/cvu025 PMC3989449

[15] M. Nahrendorf , M. J. Pittet , F. K. Swirski , Circulation 2010, 121, 2437. 20530020 10.1161/CIRCULATIONAHA.109.916346 PMC2892474

[16] F. K. Swirski , M. Nahrendorf , J. Am. Coll. Cardiol. 2013, 62, 1902. 23973700 10.1016/j.jacc.2013.07.058

[17] T. Ben‐Mordechai , R. Holbova , N. Landa‐Rouben , T. Harel‐Adar , M. S. Feinberg , I. Abd Elrahman , G. Blum , F. H. Epstein , Z. Silman , S. Cohen , J. Am. Coll. Cardiol. 2013, 62, 1890. 23973704 10.1016/j.jacc.2013.07.057

[18] E. H. Choo , J. H. Lee , E. H. Park , H. E. Park , N. C. Jung , T. H. Kim , Y. S. Koh , E. Kim , K. B. Seung , C. Park , K. S. Hong , K. Kang , J. Y. Song , H. G. Seo , D. S. Lim , K. Chang , Circulation 2017, 135, 1444. 28174192 10.1161/CIRCULATIONAHA.116.023106

[19] J. Park , B. Kim , J. Han , J. Oh , S. Park , S. Ryu , S. Jung , J. Y. Shin , B. S. Lee , B. H. Hong , D. Choi , B. S. Kim , ACS Nano 2015, 9, 4987. 25919434 10.1021/nn507149w

[20] J. Han , Y. S. Kim , M. Y. Lim , H. Y. Kim , S. Kong , M. Kang , Y. W. Choo , J. H. Jun , S. Ryu , H. Y. Jeong , J. Park , G. J. Jeong , J. C. Lee , G. H. Eom , Y. Ahn , B. S. Kim , ACS Nano 2018, 12, 1959. 29397689 10.1021/acsnano.7b09107

[21] W. Wang , J. Chen , M. Li , H. Jia , X. Han , J. Zhang , Y. Zou , B. Tan , W. Liang , Y. Shang , Q. Xu , S. A , W. Wang , J. Mao , X. Gao , G. Fan , W. Liu , ACS Appl. Mater. Interfaces 2019, 11, 2880. 30592403 10.1021/acsami.8b20158

[22] Y. Yao , J. Ding , Z. Wang , H. Zhang , J. Xie , Y. Wang , L. Hong , Z. Mao , J. Gao , C. Gao , Biomaterials 2020, 232, 119726. 31901502 10.1016/j.biomaterials.2019.119726

[23] J. Li , Y. Shu , T. Hao , Y. Wang , Y. Qian , C. Duan , H. Sun , Q. Lin , C. Wang , Biomaterials 2013, 34, 9071. 24001992 10.1016/j.biomaterials.2013.08.031

[24] P. A. Shiekh , A. Singh , A. Kumar , ACS Appl. Mater. Interfaces 2018, 10, 3260. 29303551 10.1021/acsami.7b14777

[25] C. A. Holladay , A. M. Duffy , X. Chen , M. V. Sefton , T. D. O'Brien , A. S. Pandit , Biomaterials 2012, 33, 1303. 22078809 10.1016/j.biomaterials.2011.10.019

[26] H. Liu , Y. Yang , Y. Liu , J. Pan , J. Wang , F. Man , W. Zhang , G. Liu , Adv. Sci. 2020, 7, 1903129. 10.1002/advs.201903129 PMC7141020 32274309

[27] Q. Jiang , Z. Luo , Y. Men , P. Yang , H. Peng , R. Guo , Y. Tian , Z. Pang , W. Yang , Biomaterials 2017, 143, 29. 28756194 10.1016/j.biomaterials.2017.07.027

[28] R. H. Deng , M. Z. Zou , D. Zheng , S. Y. Peng , W. Liu , X. F. Bai , H. S. Chen , Y. Sun , P. H. Zhou , X. Z. Zhang , ACS Nano 2019, 13, 8618. 31246413 10.1021/acsnano.9b02993

[29] M. Chu , W. Hai , Z. Zhang , F. Wo , Q. Wu , Z. Zhang , Y. Shao , D. Zhang , L. Jin , D. Shi , Biomaterials 2016, 91, 182. 27031812 10.1016/j.biomaterials.2016.03.018

[30] D. W. Zheng , S. Hong , L. Xu , C. X. Li , K. Li , S. X. Cheng , X. Z. Zhang , Adv. Mater. 2018, 30, 1800836. 10.1002/adma.201800836 29782675

[31] Q. Jiang , Y. Liu , R. Guo , X. Yao , S. Sung , Z. Pang , W. Yang , Biomaterials 2019, 192, 292. 30465973 10.1016/j.biomaterials.2018.11.021

[32] J. Li , X. Liu , Z. Zhou , L. Tan , X. Wang , Y. Zheng , Y. Han , D. F. Chen , K. W. K. Yeung , Z. Cui , X. Yang , Y. Liang , Z. Li , S. Zhu , S. Wu , ACS Nano 2019, 13, 11153. 31425647 10.1021/acsnano.9b03982

[33] Y. Liu , K. Ai , X. Ji , D. Askhatova , R. Du , L. Lu , J. Shi , J. Am. Chem. Soc. 2017, 139, 856. 27997170 10.1021/jacs.6b11013 PMC5752099

[34] X. Bao , J. Zhao , J. Sun , M. Hu , X. Yang , ACS Nano 2018, 12, 8882. 30028940 10.1021/acsnano.8b04022

[35] H. Zhao , Z. Zeng , L. Liu , J. Chen , H. Zhou , L. Huang , J. Huang , H. Xu , Y. Xu , Z. Chen , Y. Wu , W. Guo , J. H. Wang , J. Wang , Z. Liu , Nanoscale 2018, 10, 6981. 29610822 10.1039/c8nr00838h

[36] T. Sun , D. Jiang , Z. T. Rosenkrans , E. B. Ehlerding , D. Ni , C. Qi , C. J. Kutyreff , T. E. Barnhart , J. W. Engle , P. Huang , W. Cai , Adv. Funct. Mater. 2019, 29, 1904833. 10.1002/adfm.201904833 PMC7017599 32055240

[37] C. Cavallini , G. Vitiello , B. Adinolfi , B. Silvestri , P. Armanetti , P. Manini , A. Pezzella , M. d'Ischia , G. Luciani , L. Menichetti , Nanomaterials 2020, 10, 1518. 10.3390/nano10081518 PMC7466405 32756369

[38] M. Kim , H. S. Kim , M. A. Kim , H. Ryu , H. J. Jeong , C. M. Lee , Macromol. Biosci. 2017, 17, 1600371. 10.1002/mabi.201600371 27906510

[39] L. P. da Silva , S. Oliveira , R. P. Pirraco , T. C. Santos , R. L. Reis , A. P. Marques , V. M. Correlo , Biomed. Mater. 2017, 12, 025010. 28181477 10.1088/1748-605X/aa5f79

[40] E. Ruvinov , S. Cohen , Adv. Drug Delivery Rev. 2016, 96, 54. 10.1016/j.addr.2015.04.021 25962984

[41] A. Hasan , A. Khattab , M. A. Islam , K. A. Hweij , J. Zeitouny , R. Waters , M. Sayegh , M. M. Hossain , A. Paul , Adv. Sci. 2015, 2, 1500122. 10.1002/advs.201500122 PMC5033116 27668147

[42] N. Landa , L. Miller , M. S. Feinberg , R. Holbova , M. Shachar , I. Freeman , S. Cohen , J. Leor , Circulation 2008, 117, 1388. 18316487 10.1161/CIRCULATIONAHA.107.727420

[43] G. B. Lim , Nat. Rev. Cardiol. 2015, 12, 443. 10.1038/nrcardio.2015.107 26149488

[44] G. T. Grant , E. R. Morris , D. A. Rees , P. J. C. Smith , D. Thom , FEBS Lett. 1973, 32, 195.

[45] K. Y. Lee , D. J. Mooney , Prog. Polym. Sci. 2012, 37, 106. 22125349 10.1016/j.progpolymsci.2011.06.003 PMC3223967

[46] M. Plotkin , S. R. Vaibavi , A. J. Rufaihah , V. Nithya , J. Wang , Y. Shachaf , T. Kofidis , D. Seliktar , Biomaterials 2014, 35, 1429. 24268664 10.1016/j.biomaterials.2013.10.058

[47] P. H. Chen , H. C. Liao , S. H. Hsu , R. S. Chen , M. C. Wu , Y. F. Yang , C. C. Wu , M. H. Chen , W. F. Su , RSC Adv. 2015, 5, 6932.

[48] R. Bao , B. Tan , S. Liang , N. Zhang , W. Wang , W. Liu , Biomaterials 2017, 122, 63. 28107665 10.1016/j.biomaterials.2017.01.012

[49] X. Meng , D. A. Stout , L. Sun , R. L. Beingessner , H. Fenniri , T. J. Webster , J. Biomed. Mater. Res., Part A 2013, 10, 1095. 10.1002/jbm.a.34400 23008178

[50] Y. Huang , X. Li , Z. Lu , H. Zhang , J. Huang , K. Yan , D. Wang , J. Mater. Chem. B 2020, 8, 9794. 33030182 10.1039/d0tb01948h

[51] F. Wu , A. Gao , J. Liu , Y. Shen , P. Xu , J. Meng , T. Wen , L. Xu , H. Xu , Adv. Healthcare Mater. 2018, 7, 1800990. 10.1002/adhm.201800990 30565899

[52] A. Saparov , V. Ogay , T. Nurgozhin , W. C. W. Chen , N. Mansurov , A. Issabekova , J. Zhakupova , Inflammation Res. 2017, 66, 739. 10.1007/s00011-017-1060-4 28600668

[53] J. Kim , H. Y. Kim , S. Y. Song , S. Go , H. S. Sohn , S. Baik , M. Soh , K. Kim , D. Kim , H. C. Kim , N. Lee , B. S. Kim , T. Hyeon , ACS Nano 2019, 13, 3206. 30830763 10.1021/acsnano.8b08785

[54] E. Vergadi , E. Ieronymaki , K. Lyroni , K. Vaporidi , C. Tsatsanis , J. Immunol. 2017, 198, 1006. 28115590 10.4049/jimmunol.1601515

[55] Y. Duan , H. Zheng , Z. Li , Y. Yao , J. Ding , X. Wang , J. R. Nakkala , D. Zhang , Z. Wang , X. Zuo , X. Zheng , J. Ling , C. Gao , Biomaterials 2020, 46, 120012. 10.1016/j.biomaterials.2020.120012 32276198

[56] X. Zhang , D. Yao , W. Zhao , R. Zhang , B. Yu , G. Ma , Y. Li , D. Hao , F. J. Xu , Adv. Funct. Mater. 2021, 31, 2009258.

[57] E. Palojoki , A. Saraste , A. Eriksson , K. Pulkki , M. Kallajoki , L. M. Voipio‐Pulkki , I. Tikkanen , Am. J. Physiol.: Heart Circ. Physiol. 2001, 280, H2726. 11356629 10.1152/ajpheart.2001.280.6.H2726

[58] X. Yan , A. Anzai , Y. Katsumata , T. Matsuhashi , K. Ito , J. Endo , T. Yamamoto , A. Takeshima , K. Shinmura , W. Shen , K. Fukuda , M. Sano , J. Mol. Cell. Cardiol. 2013, 62, 24. 23644221 10.1016/j.yjmcc.2013.04.023

[59] E. Ruvinov , I. Freeman , R. Fredo , S. Cohen , Nano Lett. 2016, 16, 883. 26745552 10.1021/acs.nanolett.5b03598

[60] C. Fan , J. Shi , Y. Zhuang , L. Zhang , L. Huang , W. Yang , B. Chen , Y. Chen , Z. Xiao , H. Shen , Y. Zhao , J. Dai , Adv. Mater. 2019, 31, 1902900. 10.1002/adma.201902900 31408234

