# Supplementary material for: Natural Melanin/Alginate Hydrogels Achieve Cardiac Repair through ROS Scavenging and Macrophage Polarization
Source: Adv Sci (Weinh). 2021 Aug 19;8(20):2100505. doi: 10.1002/advs.202100505 (PMC8529445; doi:10.1002/advs.202100505)
Supplement: Supplementary file 1 — Supporting Information [file ADVS-8-2100505-s001.pdf]

WILEY-VCH

© 2021 Wiley-VCH GmbH

ADVANCED SCIENCE
Open Access

Supporting Information

for *Adv. Sci.,* DOI: 10.1002/advs.202100505

Natural Melanin/Alginate Hydrogels Achieve Cardiac Repair through ROS Scavenging and Macrophage Polarization

Jin Zhou,Wei Liu, Xiaoyi Zhao,Yifan Xian, Wei Wu, Xiao Zhang, Nana Zhao, Fu-Jian Xu,* and Changyong Wang**

Supporting Information

Natural Melanin/Alginate Hydrogels Achieve Cardiac Repair through ROS Scavenging and Macrophage Polarization

Jin Zhou,† Wei Liu,†Xiaoyi Zhao,†Yifan Xian, Wei Wu, Xiao Zhang,Nana Zhao, Fu-Jian Xu,* and Changyong Wang**

Prof. J. Zhou, W. Liu, Prof. C. Wang, Dr. W. Wu, Dr. X. Zhang
Beijing Institute of Basic Medical Sciences, 27 Taiping Rd, Beijing 100850, PR China
E-mail: wcy2000_zm@163.com

X. Zhao, Y. Xian, Prof. N. Zhao, Prof. F. J. Xu
Key Lab of Biomedical Materials of Natural Macromolecules (Beijing University of Chemical Technology, Ministry of Education)
Beijing Laboratory of Biomedical Materials, Beijing Advanced Innovation Center for Soft Matter Science and Engineering
College of Materials Science and Engineering
Beijing University of Chemical Technology
Beijing, 100029, P. R. China
E-mail: xufj@mail.buct.edu.cn (F.J. Xu), zhaonn@mail.buct.edu.cn (N. Zhao)

†These authors contributed equally to this work.

Experimental Section

Materials: Cuttlefish were purchased from Beijing SidaokouAquaculture Market (Beijing, China). Sodium alginate, calcium gluconate, safranin O,2,2´-diphenyl-1-picryl-hydrazyl (DPPH), dihydroethylpyridine (DHE), 2’,7’-dichlorodifluorofluorescein diacetate (DCFH-DA), 4’,6-diamidino-2-phenylindole (DAPI), and collagenase IVwere supplied by Sigma-Aldrich Chemical Co, St Louis, MO. Hydrogen peroxide (H_2O_2) solution (30%) andferrous sulfate heptahydrate ($FeSO_4 \cdot 7H_2O$) were purchased from Beijing Chemical Co., China.Dulbecco’s Modified Eagle’s Medium(DMEM), Roswell Park Memorial Institute (RPMI) 1640 medium,fetal bovine serum (FBS), penicillin-streptomycin, and trypsin werepurchased from Gibco, NY, USA. The antibodies of Cx43, c-TnT, Caspase-3, TNF-α, Arg1, FAK, PI3K, Akt1, iNOS, F-actin, α-SMA, CD68, CD86, CD206 and the enzyme-linkedimmunosorbent assay (ELISA) test kits of TNF-α, iNOS, and IL-10 were purchased from Abcam (Cambridge, UK). BEZ235 were purchased from Selleck, USA.Alamar Blue assay, terminal deoxynucleotidyl transferase (TdT) dUTP nick-end labeling (TUNEL) assay kit,and tissue ROS detection kit (DHE) was purchased from Biorab Biotech.

Preparation ofMNPs/Alg Hydrogels: To prepare of MNPs/Alg hydrogels, natural melanin nanoparticles (MNPs) were firstly prepared following the previously reportedmethodwith slight modifications.[S 1]Briefly,MNPs were extracted from the dissectedcuttlefish ink sac,followed by centrifugation at 12000 rpm for 10 min to remove large particles and 18000 rpm at 4 ^{o}C for 10 min. MNPs were washed with deionized water for three times and suspended in deionized water for further use. For the preparation ofMNPs/Alg hydrogels, differentamounts of MNPs were added to the sodium alginate solution(3wt%). The final concentration of MNPs was 0, 0.2, 0.5, 1.0,and 2.0 mg/mL, respectively. Then, calcium gluconate solution (3 wt%) was added dropwise into the MNPs/Alg solution (volume ratio: 1:3) under stirring. Finally, MNPs/Alg hydrogels can be formed within 10 min.

Characterization of MNPs and MNPs/Alg Hydrogels: Transmission electron microscopy (TEM), scanning electron microscope (SEM),atomic force microscopy (AFM) androtational rheometer were used to characterize MNPs and MNPs/Alg hydrogels. For TEM and SEM specimen preparation, the samples in aqueous solution were dropped onto a Formvar-covered copper grid or a silicon wafer, followed by drying naturally. A Tecnai G2 analytical electron microscope (FEI Company, Hillsboro, OR) and a field emission SEM (Zeiss Supra55) were employed for TEM and SEM measurements, respectively.Atomic force microscopy (AFM) studies werecarried out with the Dimension Icon model with a NanoscopeIIIa controller (Bruker, Santa Barbara, CA).The rheological properties of hydrogels were testedusing stress-controlled rheometer (HR-1, TA Instrument, USA) with 20 mm flat plates.

*ROS Scavenging by MNPs/Alg Hydrogels:*The antioxidant activity of MNPs/Alg hydrogels was characterized by hydrogels to scavenge hydroxyl radicals (·OH) and DPPH. ·OH was generated via Fenton reactions according to the previous report with minor modifications.[S2] Briefly, $FeSO_4$ solution (2 mM, 600 μL) and Safranin O solution (360 μg/mL, 500 μL) were mixed, which were mixed with300 μL of as-prepared MNPs/Alg hydrogels. For the blank group and control group, deionized water (300 μL) was employed instead of MNPs/Alg hydrogels. After 10 min, 800 μL of H_2O_2 (0.06 wt%) or water (as the control group) was added into the mixed solution and inculcated at 55 ^{o}C for 60 min.The absorbance of the reaction mixtures was measured at 492 nm and the scavenging effect of MNPs/Alg hydrogels on ·OH was calculated according to eq 1.

To test the scavenging effect on DPPH radicals, 300 μL ofhydrogels or water (control group) was immersed in 800 μL of DPPH solution (0.1 mM, in 95wt% ethanol) for 6 h in dark, the OD (optical density) value at 517 nm of the mixture was measured using a microplate reader (BioTekCytation 3), and the scavenging effect on DPPH radicals was obtained using eq 2.

$$\mathbf{Scavenging\ Effect(\%)} = \mathbf{100} \times \frac{\mathbf{A_{sample}} - \mathbf{A_{blank}}}{\mathbf{A_{control}} - \mathbf{A_{blank}}} \qquad (1)$$

$$\text{Scavenging Effect}(\%) = 100 \times \frac{A_{control} - A_{sample}}{A_{control}} \qquad (2)$$

*Isolation of Cardiomyocytes, Bone Marrow-Derived Macrophages and Cell Culture:*Cardiomyocytes (CMs) were isolated from 0- to 24 h- old newborn Sprague Dawley (SD) rats. Ophthalmic scissors were used to cut the chest laterally to expose the heart, and the whole heart was removed from the aortic root and placed in 4°Csterile PBS to remove blood. The atrial appendage was removed with forceps, and the remaining parts were cut into pieces. Subsequently, the minced tissues were digested by trypsin (0.05%) and collagenase IV(0.25%) at 37°C at a rate of 5 min/timeto obtain a single cell suspension. The digestion suspension was collected in a high-glucose DMEM medium containing 15% fetal bovine serum (FBS) and centrifuged at 1300 rpm for 7 min. The supernatant was discarded after centrifugation. The cells were resuspended in DMEM medium containing 15% FBS.Finally, after 2 h of differential adherence, the supernatant was pipetted to obtaincardiomyocytes.

Bone marrow-derived macrophages (BMDMs) were isolated from C57BL/6 mice as previously reported.[S3,S4] Briefly, the femur and tibia were separated aseptically, and the bone marrow cells were washed out with RPMI-1640 medium and centrifuged at 300 g for 5 min to obtain cell pellets. Subsequently, the cells were cultured in RPMI-1640 medium containing 20 ng/mL macrophage-colony stimulating factor (M-CSF), 2 mM L-glutamine, 10% FBS, and 1% penicillin-streptomycin in a petri dish. After 8 d, flow cytometry was used to detect F4/80 to analyze the purity of the macrophages. On day 9, BMDMs were collected and then stimulated by M1 macrophages polarization stimulating solution containing LPS (100 ng/mL) and IFN-γ (20 ng/mL).

Primary human umbilical vein endothelial cells (HUVECs) were purchased from Lonza (Walkersville, MD, USA) and cultured as described in the manufacturer's instructions.

Viability of Cardiomyocytes with the Hydrogels and MNPs: MNPs/Alg hydrogels with different MNP concentrations (0, 0.2, 0.5, 1, 2 mg/mL) were added to the culture medium of

neonatal rat CMs after 3 days of primary isolation culture. Then the cells were cultured for 3, 7, and 10 days continuously. The CMs cultured medium was removed and replaced with 10% Alamar Blue solutions. After being cultured for another 4 h, the absorbance of the supernatant at 570 and 612 nm was measured by a microplate reader. Next, 200 μM H_2O_2 was introduced to the culture medium for 3 days to induce oxidative stress. The viability of CMs in the ROS microenvironment was further assessed by Alamar Blue assay.The viability of CMs with free MNPs was studied after the incubation of neonatal rat CMs withMNPs (0, 0.2, 0.5, 1, 2 mg/mL) for 1 or 3 days continuously. The CMs cultured medium was removed and replaced with 10% Alamar Blue solutions. After being cultured for another 4 h, the absorbance of the supernatant at 570 and 612 nm was measured by a microplate reader.

Dihydroethidium (DHE) and 2',7'-dichlorofluorescin diacetate (DCFH-DA) staining were used to evaluate the intracellular superoxide anions level and the amount of a variety of ROS, respectively. Briefly, CMs were cultured in normal or ROS microenvironment induced by 200 μM H_2O_2 with different MNPs/Alg hydrogels (concentration of MNPs: 0, 0.2, 0.5, 1, and 2 mg/mL). The cells were incubated with DHE (5 mM) or DCFH-DA (10 μM) for 15-25 min in darkon Day 1 and 3, respectively. After being washed with PBS, CMs were stained by 4',6-diamidino-2-phenylindole (DAPI). The samples were observed under confocal laser scanning microscope (CLSM, Nikon Ti A1). The relative fluorescence intensity of DHE and DCFH-DA was quantified using IPWIN60 software (Media Cybernetics, Inc.). At the same time, the above CMs were detected using the apoptosis detection kit to detect apoptosis in the absence and presence of ROS.

*In Vitro Degradation Assay:*The degradation of the MNPs/Alg hydrogel was investigatedfollowing the previous report.[S5]200 μL of the hydrogel([MNPs]: 1 mg/mL) were incubated in DMEM (2 mL) at 37 °Cfor 21 days. After differenttime periods (1, 3, 5, 7, 10, 14, 17, and 21 days), the samples were driedcompletely and weighed. Then the percentage of the mass loss was calculated. Three samples were used for each time period.

*Assessment of Macrophage Polarization in Vitro:*MNPs/Alg hydrogel was added to the culture medium of BMDMs under ROS microenvironment with or without LPS for 24 h. Relative expressions of pro-inflammatory (TNF-α, CCL-2 andiNOS) or anti-inflammatory (IL-10, TGF-β and Arg1) genes and proteins were evaluated using qRT-PCR, immunofluorescence staining assay, Western blot and ELISA.

Western Blotting Analysis of MNPs/Alg hydrogel on Macrophage Polarization through the PI3K/Akt1/mTOR Axis: PBS, Alg hydrogel or MNPs/Alg hydrogel were added to the culture medium of BMDMs under ROS microenvironment. The BMDMs were washed twice with cold PBS and then lysed on ice for 10 min. The supernatant was collected after centrifugation at 12000 rcf for10-15 min. The extracted proteins were quantified using BCA ProteinAssay Kit (Thermo Scientific, USA). The extracted proteins were loaded on a 15% sodium dodecyl sulfate (SDS)-polyacrylamide gels, separated by electrophoresis. Then they were transferred to polyvinylidene difluoride (PVDF) membranesand sequentially incubated with primary antibodies and secondaryantibodies. The primary antibodies of focal adhesion kinase (FAK),phosphorylated FAK (p-FAK), phosphoinositide 3-kinase (PI3K), p-PI3K, v-akt murine thymoma viral oncogenehomolog 1 (Akt1) and p-Akt1 and GAPDH (Abcam, Cambridge, UK). After being washed with Tris-base buffer, they were furtherincubated using appropriate secondary antibodies (goat antimouse orantirabbit IgG) for 1 h at room temperature. Finally, the membranes were imaged with an enhanced chemiluminescentreagent (Applygen, Beijing, China)and chemiluminescence imaging system.

*Inhibition Experiments:*The PI3K/mTOR inhibitor (BEZ235, Selleck, USA) were added into the culture medium of BMDMs with 200 μM H_2O_2. After 48 h, BMDMs in different groups were harvested for qRT-PCR to detect the expression of M2-related genes.

Quantitative Real-Time Polymerase Chain Reaction Analysis: Total RNA from the MI region in different groups was extracted using the TRIzol method. RNA fromeach sample was

reverse-transcribed into cDNA using the Reverse Transcription kit (Toyobo, Japan). The qRT-PCR was performed with the SYBR® Premix Ex Taq ™ II Kit (Toyobo, Japan). Primers were designed based on the cDNA sequence in NCBI. GAPDH mRNA was used as an internal reference for detection. The primer sequences are listed in Table 1. The qRT-PCR reaction system includes: 2 μL of cDNA, 2 μL of primer (1 μmol/L), 10 μL of SYBR green dye (2×), and 6 μL of Nuclease-Free H_2O. The total reaction system is 20 μL. Reaction conditions: 95 ^{o}C for 30 s, 95 ^{o}C for 3 s, 55 ^{o}C for 30 s, 72 ^{o}C for 30 s, 95 ^{o}C for 60 s, 55°C for 30 s, and 95°C for 30 s. 42 amplification cycles were performed. The PCR was confirmed by the product melting curve, and the Ct values of the target gene and GAPDH of the sample were obtained based on the PCR curve. The relative expression level for target genes were performed with a value of $2^{-\Delta\Delta Ct}$.

Table S1: Sequence of Primers for q-PCR.

Genes	Forward primer	Reverse primer
c-TnT	TGAACAGCAGCGTATTCG	GACAGAGCCTTCTTCTTCC
cx43	AGCAAGCTAGCGAGCAAAAC	GAGTTCATGTCCAGCAGCAA
caspase-3	ATGGAGAACAACAAAACCTCAGT	TTGCTCCCATGTATGGTCTTTAC
TNF-*α*	GGCTCCCTCTCATCAGTTCC	CGCTTGGTGGTTTGCTACG
iNOS	AACAACGTGGAGAAAACCCCA	GGGTCGATGGAGTCACATGC
CCL-2	CTCTTGAGCTTGGTGACAAATACT	CGGCTGGAGAACTACAAGAGA
TGF-*β*	CTGCTGACCCCCACTGATAC	CTGTATTCCGTCTCCTTGGTTC
IL-10	GCTGTCATCGATTTCTCCCCT	AGATGTCAAACTCATTCATGGCC
Arg1	CATATCTGCCAAGGACATCG	GGTCTCTTCCATCACTTTGC
VWF	ATGGTTCTGGATGTGGCGT	TTGCTCCTGTTGAAGTCGG
Vcam1	TTCCCTAGAGATCCAGAAATCGAG	CTTGCAGCTTACAGTGACAGAGC
CD31	CACTTCTGAACTCCAACAACG	GGACACTTGAACTTCCGTG

*In Vivo Degradation Assay of Hydrogel:*Sprague-Dawley rats (250 ± 10 g) were used for the evaluation of the *in vivo* degradation of Alg and MNPs/Alg hydrogels in the subcutaneous and heart regions, respectively. The degradation in the subcutaneous site was investigated following the previous report.[S6]After anesthesia, two incisions (2 cm in length) were made on the tope sides of the back. The skin was cut and 100 mg of hydrogel was implanted into each subcutaneous pocket. At 1, 7 and 14day, the and MNPs/Alghydrogels with the surrounding tissues were collected from subcutaneous to measure their weight. The weight change of hydrogel remaining in the subcutaneous site was then calculated.

Myocardial Infarction Model and Treatment: Male SD rats (250 ± 10 g) were used to prepare the MI animal model as previously reported.[S7,S8]The rats were anesthetized by intraperitoneal injection of 2% sodium pentobarbital.The heartwas exposedfrom the left thoracotomy and the proximal left anterior descending artery (LAD) was ligated with 6-0 polypropylene. The success of MI was confirmed by the typical STsegment elevation in electrocardiography. The MI model rats were randomly divided into 4 groups (n = 10 each group) as follows, (1) sham (no MI, underwent thoracotomy only without LAD ligation); (2) PBS (100 µL); (3) Alg hydrogel (100 µL); (4) MNPs/Alg hydrogel (100 µL). The hydrogelswere rapidly injected in the border of the infarct area at three differentlocations with a 28-gauge needle.Then, chest was closed in multiple layers. Rats were sacrificed on Days 1, 3, 5, and 28, respectively after different treatments. Heart tissues were obtained foranalysis. All animal experimentalprocedures in this study were done according to Guide for the Careand Use of Laboratory Animals published by the US NationalInstitutes of Health (NIH Publication, eighth Edition, 2011) andapproved by the Institutional Animal Care and Use Committee(IACUC) of the Chinese Academy of Military Medical Science(Beijing, China).

In Vivo Measurement of Intracellular ROS in Rat Infarct Model: For measurement of intracellular ROS *in vivo*, DHE staining was performed. Briefly, the heart sections from the MI area were collected at Day 1 and Day 3, respectively after different treatments, and then

the sections in different groups were incubated with DHE (5 mM) for 15-25 min in the dark. After being washed with PBS, the heart sections were counterstained by DAPI, and examined under CLSM (Nikon Ti A1).ROS detection kit (DHE) was used to quantitatively measure DHE content in heart tissue. Briefly, fresh heart tissue was obtained at Day 1 and Day 3, respectively and washed with PBS. 50 mg of tissue from each group was weighed accurately and 1 mL of homogenization buffer were added. The mixture was homogenized with a glass homogenizer and then centrifuged at 1000 g for 10 min at 4 ^{o}C to collect the supernatant. Next, the protein concentration of the tissue supernatant is detected. The supernatant and DHE probe were added according to the protocols and incubated at 37 ^{o}C for 30 min in the dark. Finally, the fluorescence intensity was detected at an excitation wavelength of 535 nm and an emission wavelength of 610 nm using a microplate reader.

Immunohistochemistry for the Assessment of Inflammation, Apoptosis, and Angiogenesis: To evaluate the anti-inflammatory and anti-apoptosis effect after treatments, heart samples from MI areas were collected on Day 1, 3, 5 and 28, respectively. Briefly, for anti-inflammatory assessment, heart sections from different groups were fixed in 4 wt% paraformaldehyde solution for 24 h, dehydrated by gradient concentration of ethanol, and transparent by xylene. The samples were cut into sections with 4 μm thickness on the slides. For immunofluorescence staining, heart sections were rehydrated in ascending concentrations of ethanol, citrate buffer for 10 min at microwave high temperature, permeabilized in 0.3 % Triton X-100 for 30 min, and blocked with 1% (w/w) bovine serum for 30 min. The heart sections were incubated with primary antibodies overnight at 4 ^{o}C for the detection of M1 macrophages (rabbit anti-CD86 and rabbit anti-TNF-α, Abcam) and M2 macrophages(rabbit anti-CD206 and rabbit anti-Arg1, Abcam). Subsequently, the sections were washed with PBS and then incubated with Cy3-labeled secondary antibodies (goat anti-rabbit IgG, BosterBio) for 90 min. Finally, cell nuclei were counterstained with DAPI. For anti-apoptosis evaluation,

the heart sections were treated by TUNEL assay. To evaluate the neovascularization, anti-α-smooth muscle actin (α-SMA) and anti-cardiac Troponin T (c-TnT) antibodies were used to incubate with the heart sections. All the heart sections were visualized using a confocal laser-scanning microscope (A1; Nikon, Tokyo, Japan).

Histological Analysis: To analyze the infarct size and wall thickness of the MI zone, the hearts from different groups were collected onDay 28 and the embedded heart sections were prepared. The samples from the ligation point to the apex of the heart were stained by Masson's trichrome staining, and then the images were analyzed by Image J.

Echocardiography for Evaluation of Cardiac Functions: The functions of left ventricular (LV) were investigated by echocardiograms from different groups at day 28 after surgery. MI model rats were anesthetizedand performed echocardiogram on a 13 MHz linear ultrasonic transducer (15L8; Acuson Corporation, Mountain View). Left ventricle End-systolic diameter (LVIDS), left ventricular diameter End-diastole (LVIDD), left ventricular ejection fraction (LVEF) and fractional shortening (LVFS) were analyzed according to the previous report.[S8]

*Statistical Analysis:*All data are presented as mean ± SD and are from at least three independent experiments ($n \geq 3$). Statistical significance was calculated by using Student's *t* test when two groups were compared. Statistical analyses were performed with Origin Pro 8.5 software. *$p < 0.05$ or **$p < 0.01$ was considered statistically significant.

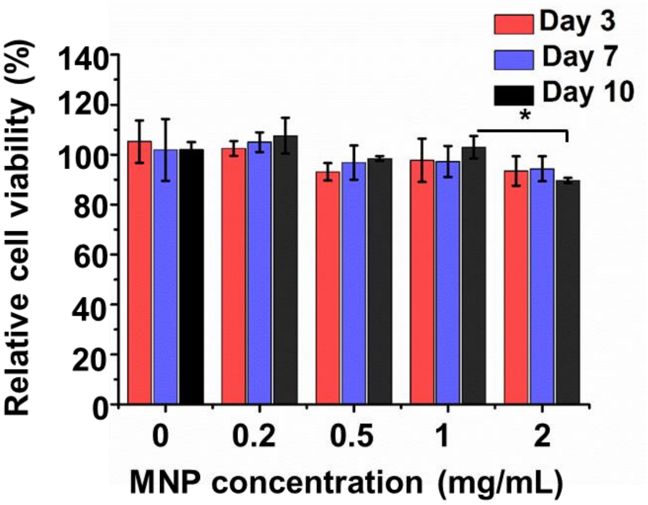

Figure S1. Viability of CMs cultured withMNPs/Alg hydrogels withdifferentMNP concentrations for 3, 7, and 10 days, respectively.(mean ± SD, n = 3, *p< 0.05, Student's t test)

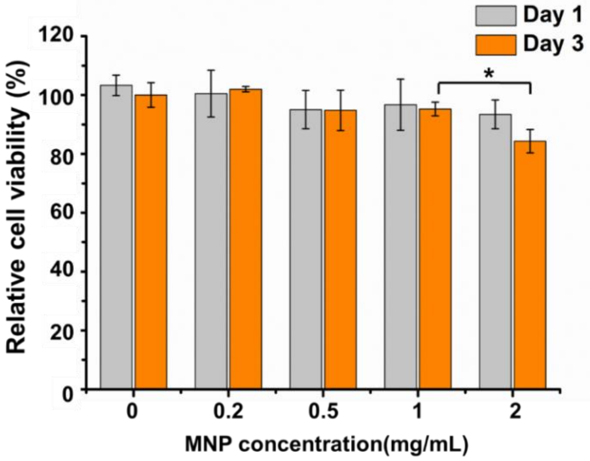

Figure S2. Viability of CMs cultured with MNPs with free MNPs with different concentrations for 1 and 3 days, respectively.(mean ± SD, n = 3, *p< 0.05, Student's t test)

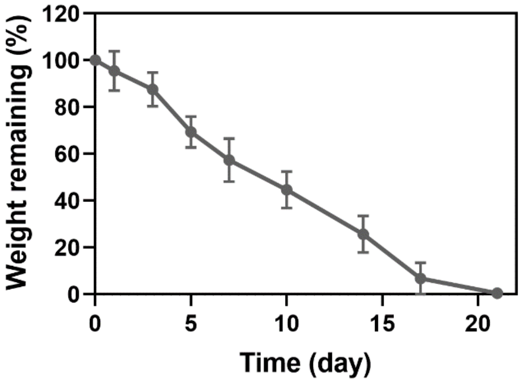

Figure S3. The *in vitro* degradation kinetics of the MNPs/Alg hydrogelover 21 days.(mean ± SD, n = 3).

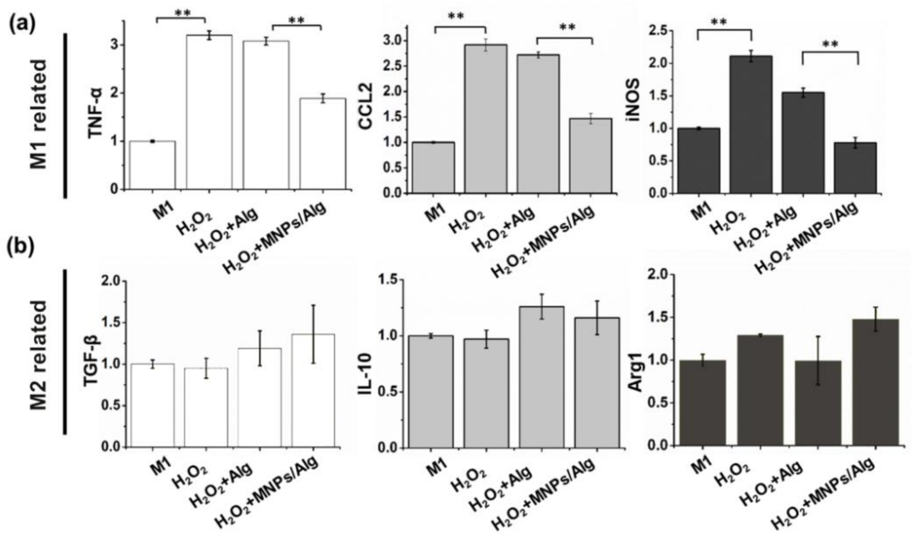

Figure S4.BMDMs were pretreated with LPS for 24 h to generate M1 phenotype and then treated with Alg or MNPs/Alg hydrogels in the ROS microenvironment.Gene expression of a) TNF-*α*, CCL-2, and iNOS, as well as b) IL-10, TGF-*β*and Arg1 analyzedby qRT-PCR.(mean ± SD, $n = 3$, $^{**}p< 0.01$, Student's *t* test).

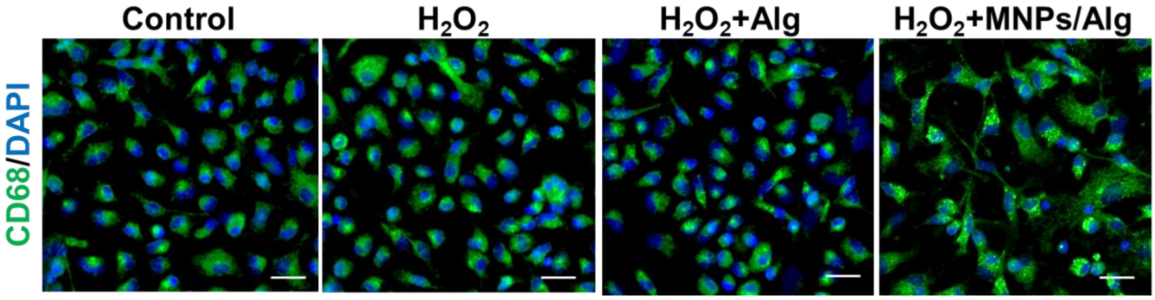

Figure S5. Representative immunofluorescence images showing obvious morphological changes of BMDMs after different treatments. Scale bar: 50 μm.

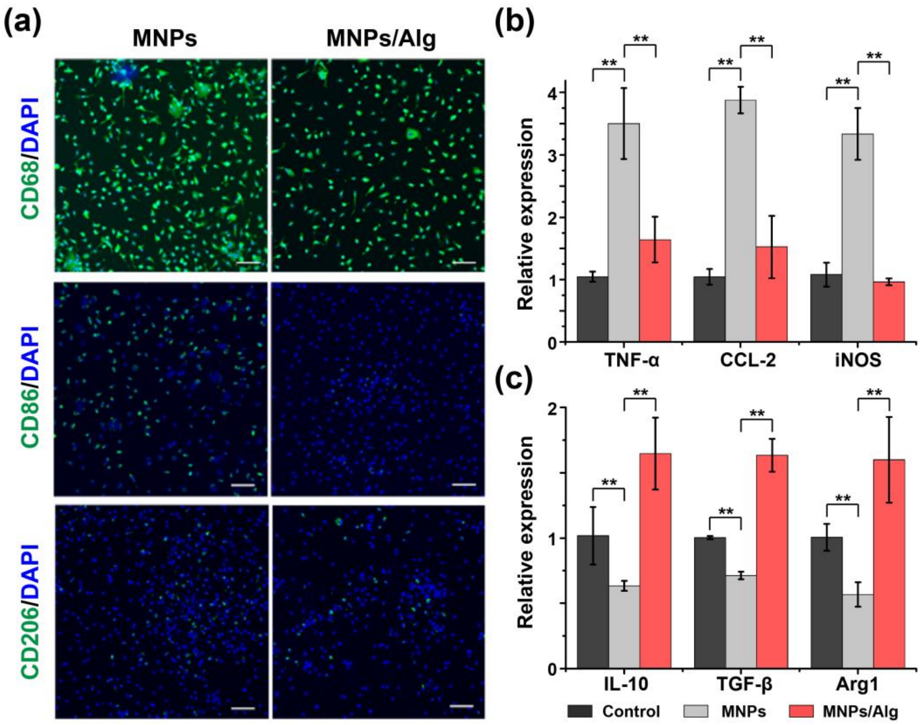

Figure S6. Effect of MNPs and the MNPs/Alg hydrogel on polarization of macrophages in the ROS microenvironment. a) Representative immunofluorescence images of CD68, CD86 and CD206 with different treatments in BMDMs for 1 day. qRT-PCR assay of the expression of b) pro-inflammatory genes (TNF-α, CCL-2, iNOS) and c) anti-inflammatory genes (IL-10, TGF-β and Arg1) in BMDMs treated with MNPs for 1 day. Scale bar: 100 μm. (mean ± SD, n = 3, **p < 0.01, Student's t test).

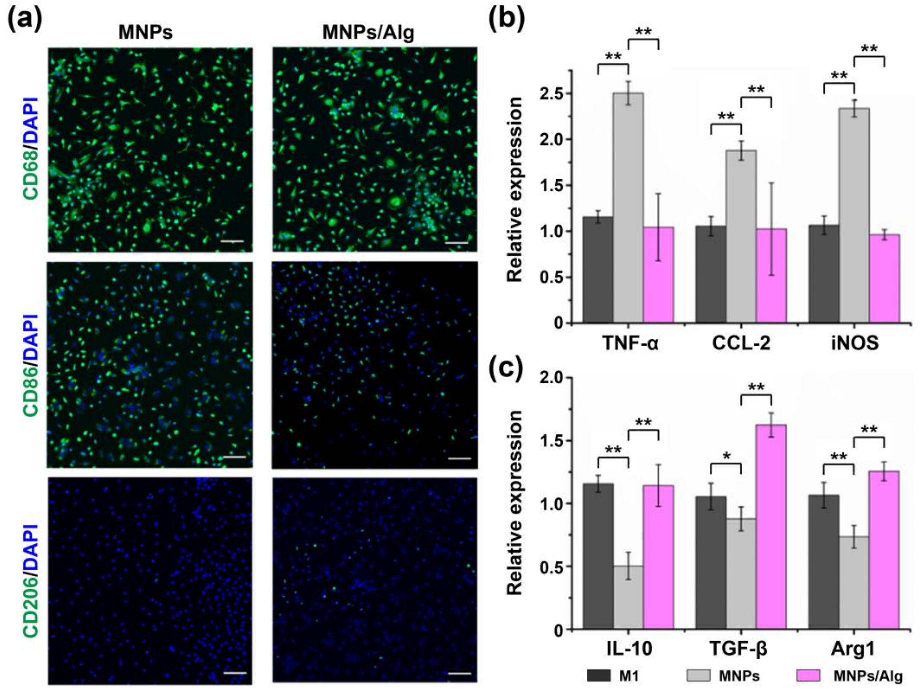

Figure S7.BMDMs were pretreated with LPS for 24 h to generate M1 phenotype and then treated with MNPs and the MNPs/Alg hydrogel, respectively. a) Representative immunofluorescence images of CD68, CD86 and CD206 with different treatments in BMDMs for 1 day. Gene expression of b) TNF-*α*, CCL-2, and iNOS, as well asc) IL-10, TGF-*β*and Arg1 analyzedby qRT-PCR.(mean ± SD, *n* = 3, ***p*< 0.01, Student's *t* test).

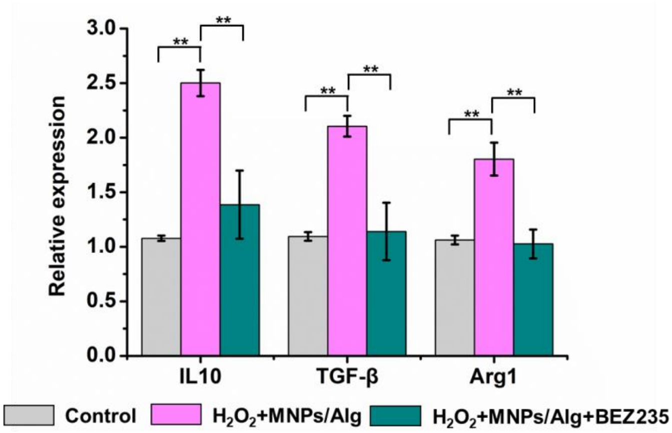

Figure S8. Expression of M2-relatedgenes of BMDMs being cultured with MNPs/Alg hydrogel with or without BEZ235 (200 ng mL^{-1}) for 48 h.(mean ± SD, n = 3, **p< 0.01, Student's t test).

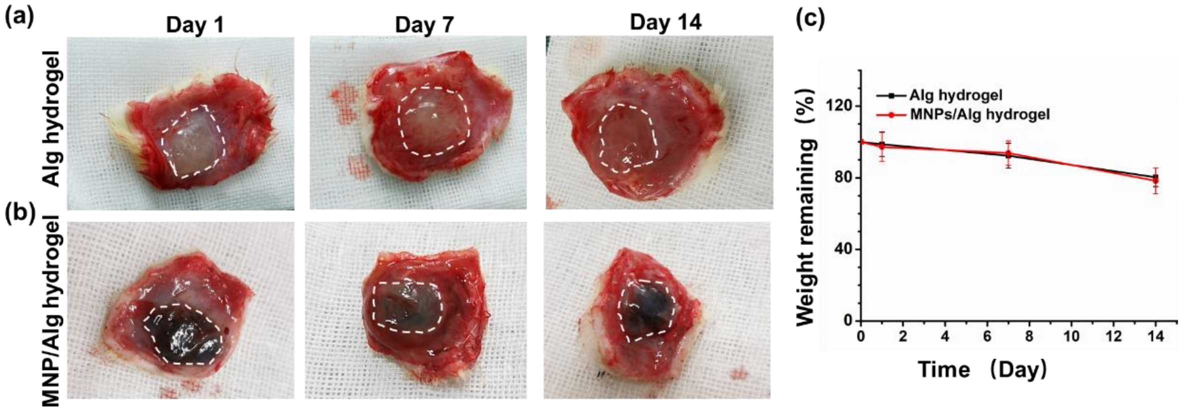

Figure S9.In $vivo$ degradation of the Alg and MNPs/Alghydrogels, respectively. Representative photographs of subcutaneous tissue with the Alg (a) and MNPs/Alg (b) hydrogel on Days 1, 7, and 14. (The hydrogels were marked by white dotted lines). b) Quantified weight remaining of the Alg and MNPs/Alg hydrogels (n = 3).

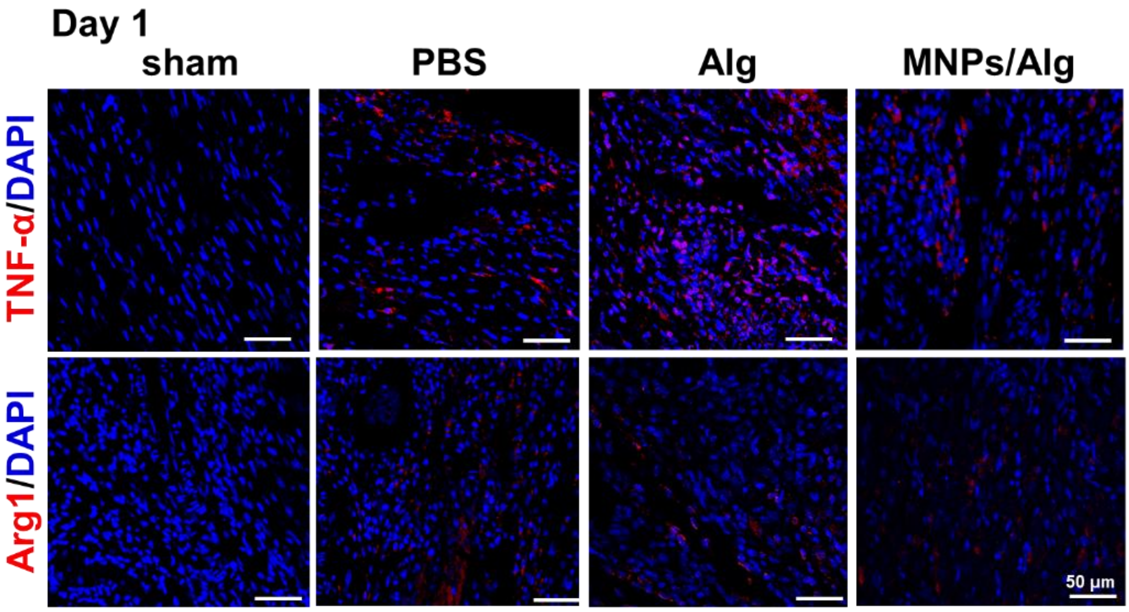

Figure S10. Immunofluorescence images of TNF-*α* (M1 macrophages related) and Arg1 (M2 macrophages related) in MI region 1day after treatments.

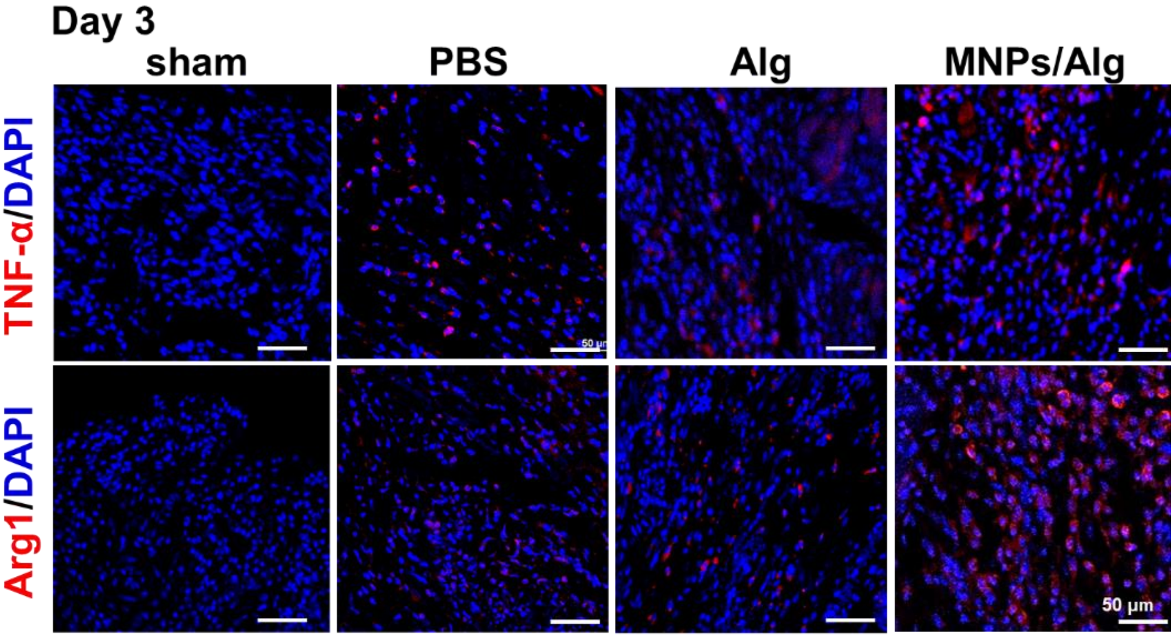

Figure S11. Immunofluorescence images of TNF-*α* (M1 macrophages related) and Arg1 (M2 macrophages related) in MI region 1day after treatments.

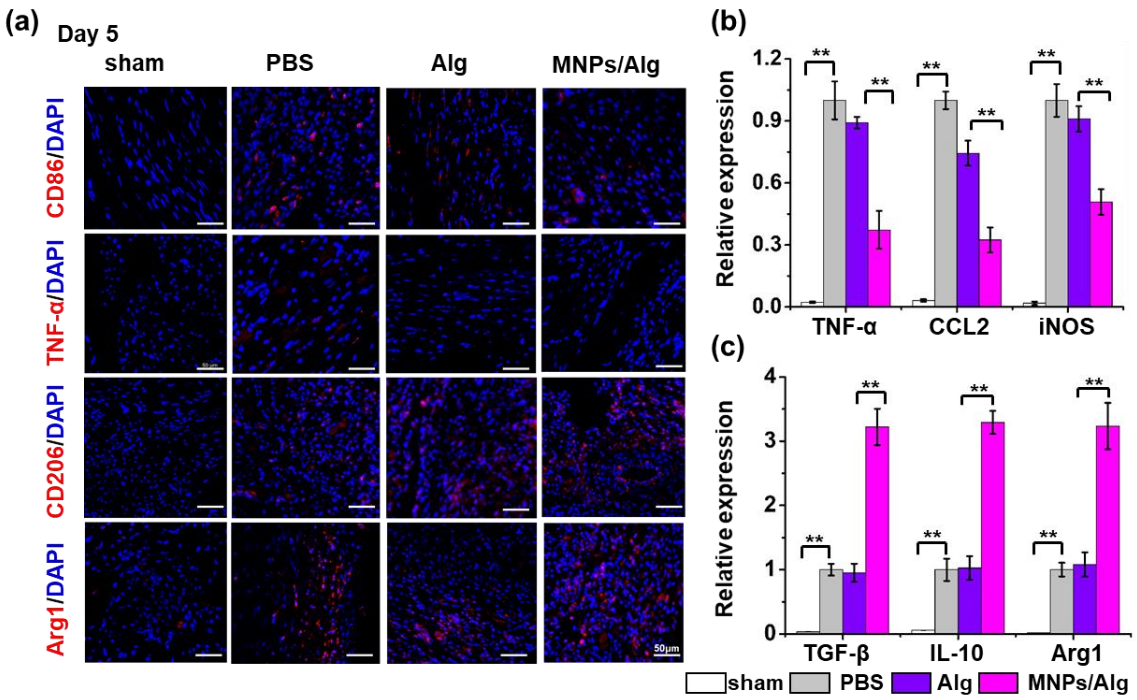

Figure S12. a)Immunofluorescence images of CD86, TNF-α, CD206, and Arg1 in the MI region on Day 5.b) qRT-PCR analysis of M1-related (TNF-α, CCL2 and iNOS) and c) M2-related (TGF-β, IL-10 and Arg1) gene expression in MI region on Day 5.(mean ± SD, n = 3, **p< 0.01, Student's t test)

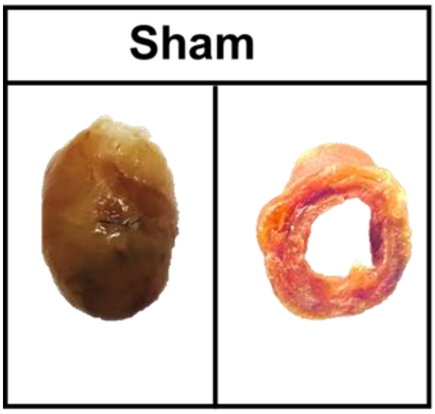

Figure S13. Photographs of the heart and TTC-stained heart section of Sham group after 28 days.

References

[S 1]Q. Jiang, Z. Luo, Y. Men, P. Yang, H. Peng, R. Guo, Y. Tian, Z. Pang, W. Yang,*Biomaterials* **2017,***143*, 29.

[S2]T. Hao, J. Li, F. Yao, D. Dong, Y. Wang, B. Yang, C. Wang,*ACS Nano***2017**, *11*, 5474.

[S3]J. Weischenfeldt, B. Porse, *CSH Protoc 2008*, **2008**, pdb.prot5080.

[S4]L. Zhu, T. Yang, L. Li, L. Sun, Y. Hou, X. Hu, L. Zhang, H. Tian, Q. Zhao, J. Peng, H. Zhang, R. Wang, Z. Yang, L. Zhang, Y. Zhao,*Nature Commun.***2014**, *5*, 4696.

[S5] Y. Li, X. Chen, R. Jin, L. Chen, M. Dang, H. Cao, Y. Dong, B. Cai,G Bai, J. J. Gooding, S. Liu, D. Zou, Z. Zhang, C. Yang, *Sci. Adv.***2021**, *7*, eabd6740.

[S6] X. Zhang, D. Yao, W. Zhao, R. Zhang, B. Yu,G. Ma, Y. Li, D. Hao, F. J. Xu, *Adv. Funct. Mater.***2021**, *31*, 2009258.

[S7]Y. Miyahara, N. Nagaya, M. Kataoka, B. Yanagawa, K. Tanaka, H. Hao, K. Ishino, H. Ishida, T. Shimizu, K. Kangawa, S. Sano, T. Okano, S. Kitamura, H. Mori,*Nat.Med.***2006**, *12*, 459.

[S8]Y. Shu, T. Hao, F. Yao, Y. Qian, Y. Wang, B. Yang, J. Li, C. Wang,*ACSAppl. Mater. Interfaces***2015**, *7*, 6505.
